# Remuscularization with triiodothyronine and β_1_-blocker therapy reverses post-ischemic left ventricular dysfunction and adverse remodeling

**DOI:** 10.1038/s41598-022-12723-2

**Published:** 2022-05-25

**Authors:** Nikolay Bogush, Lin Tan, Emmen Naqvi, John W. Calvert, Robert M. Graham, W. Robert Taylor, Nawazish Naqvi, Ahsan Husain

**Affiliations:** 1 Division of Cardiology, Department of Medicine, Emory University School of Medicine, 3311 WMRB, 323 WMRB, 101 Woodruff Circle, Atlanta, GA 30322 USA; 2 Department of Surgery, Carlyle Fraser Heart Center, Emory University School of Medicine, Atlanta, GA 30322 USA; 3 Victor Chang Cardiac Research Institute, Sydney, NSW 2010 Australia; 4 Cardiology Division, Atlanta Veterans Affairs Medical Center, Decatur, GA 30033 USA; 5 Department of Biomedical Engineering, Emory University School of Medicine and Georgia Institute of Technology, Atlanta, GA 30322 USA

**Keywords:** Cardiac regeneration, Myocardial infarction, Heart failure

## Abstract

Renewal of the myocardium by preexisting cardiomyocytes is a powerful strategy for restoring the architecture and function of hearts injured by myocardial infarction. To advance this strategy, we show that combining two clinically approved drugs, but neither alone, muscularizes the heart through cardiomyocyte proliferation. Specifically, in adult murine cardiomyocytes, metoprolol, a cardioselective β_1_-adrenergic receptor blocker, when given with triiodothyronine (T3, a thyroid hormone) accentuates the ability of T3 to stimulate ERK1/2 phosphorylation and proliferative signaling by inhibiting expression of the nuclear phospho-ERK1/2-specific phosphatase, dual-specificity phosphatase-5. While short-duration metoprolol plus T3 therapy generates new heart muscle in healthy mice, in mice with myocardial infarction-induced left ventricular dysfunction and pathological remodeling, it remuscularizes the heart, restores contractile function and reverses chamber dilatation; outcomes that are enduring. If the beneficial effects of metoprolol plus T3 are replicated in humans, this therapeutic strategy has the potential to definitively address ischemic heart failure.

## Introduction

Approximately seven million adults in the United States have heart failure^1^, which in most cases is due to ischemic myocardial injury^2^. Severe forms of heart failure are refractory to guideline-directed medical therapies, such as angiotensin inhibitors, β-adrenergic receptor (β-AR) blockers and diuretics; heart transplantation being the only definite treatment^3^. However, demand for donor hearts far exceeds supply, so that three in four patients with severe ischemic heart failure die within one year of diagnosis^4^. Severe ischemic heart failure, therefore, remains an important public health problem and an unmet need for novel therapies to improve cardiac repair and function and, hence, outcomes.

Therapies that re-activate CM proliferation in adult hearts would potentially address the sequelae of severe ischemic heart failure by rebuilding heart muscle and reversing adverse remodeling^5^. These therapies are mostly directed at reversing the mechanism(s) by which post-neonatal CMs become cell cycle arrested. However, such approaches face enormous technical and economic barriers that impede their effective translation into treatments for severe postischemic heart failure. For example, many proposed therapies involve intramyocardial administration of viral constructs^6–9^ or miRs^10^, which are confounded by issues such as immunogenicity, toxicity and transduction in off-target cells^11^. Moreover, because of the invasive nature of delivery (intramyocardial injections), such therapies are technically challenging and limited to administration at major medical centers, and are expensive. Still other regenerative therapies are unsafe because they elicit uncontrolled CM proliferation^12^. New approaches, therefore, are needed to develop regenerative therapies that are safe, have little or no toxicity, lend themselves to non-invasive administration and induce sufficient, but not excessive, CM proliferation.

During the neonatal period (up to postnatal day 6 (P6)^13^), murine ventricular cardiomocytes (CMs) proliferate under basal conditions^14,15^ and, importantly, proliferate at a higher rate after myocardial injury^16^. While it is traditionally thought that basal CM proliferation slowly decreases from birth to P6^15,17^, temporal studies show that the CM endowment does not increase between birth and P4, but increases rapidly between P4 and P5^18^. This is associated with an increase in ventricular cyclin A2 expression^19^, which is implicated in the initiation and progression of DNA synthesis, and incorporation of^3^H-thymidine^20^, which mark S phase. While many CMs that enter the S phase complete the cell cycle and give rise to two daughter cells, which increases CM endowment—CM number increase by about 30% during the late neonatal period^18^—others fail to complete cytokinesis, but rather undergo acytokinetic mitosis, which results in binucleation^20^.

In cycling CMs, cytokinesis is partially inhibited by β-ARs because their activation represses cell cycle-associated expression of epithelial cell transforming gene 2 (ECT2), which is essential for cytokinesis^21^. Non-selective β-AR blockade during the neonatal period decreases the proportion of binucleated CMs by ~ 20% and increases CM numbers by ~ 20%^21^. However, β-AR blockers are incapable of inducing cell cycle reentry in deeply quiescent adult CMs^21^. We hypothesized that, in the setting of the long-term injured adult heart, a β-AR blocker in combination with a CM mitogen could increase cytokinesis in cycling CMs, which is necessary for remuscularization. Such a therapeutic approach has significant translational significance, since β-AR blockers are an essential component of guideline-directed medical therapy for ischemic heart failure.

In many regenerative strategies, growth factor signaling is used to increase CM proliferation^5^. Central to their proliferative signaling is stimulation of mitogen-activated protein kinase kinases 1 and 2 (MEK1/2)-mediated extracellular signal-regulated kinases 1 and 2 (ERK1/2) phosphorylation and nuclear translocation^22,23^. However, the use of growth factors to stimulate CM proliferation in adults needs to overcome two key hurdles. First, immediately after the neonatal period, expression of many growth factor receptors is repressed in ventricular CMs, making them unresponsive or under-responsive to growth factor signaling. This is the main reason why, in some regenerative strategies, constitutively active growth factor receptor overexpression (e.g., ERBB2, receptor for neuregulin-1) has been used to stimulate CM proliferation^17^. Second, expression of the nuclear phospho-ERK1/2-specific phosphatase, dual-specificity phosphatase-5 (DUSP5), is activated during postnatal development, which limits growth factor-induced increases in nuclear phospho-ERK1/2^19^.

The maturational effects of triiodothyronine (T3, a thyroid hormone) on CMs are well known^24,25^. However, T3 also stimulates proliferation of neonatal murine CMs *in vivo*^18^. The mechanism involves an increase in the transcription and synthesis of insulin-like growth factor-1 (IGF-1) and IGF-1 receptor (IGF-1R)^18,19^ and subsequent phosphorylation and nuclear localization of ERK1/2^19^. This action of T3 is cell autonomous^18^. The ability of T3, given parenterally, to simultaneously increase IGF-1 and its cognate receptor in adult CMs^19^ overcomes a key hurdle in a therapeutic strategy to rebuild heart muscle. However, its ability to stimulate CM proliferation in adult hearts is attenuated by the maturational acquisition of DUSP5 expression in CMs^19,26^. In many cells other than CMs^19^, DUSP5 acts as a negative feedback regulator of nuclear ERK1/2 signaling^27^. Not surprisingly, therefore, DUSP5 is a tumor suppressor^28^ and it is also essential for T cell survival^29^. Its global inhibition, therefore, could be problematic. We hypothesized that β-AR blockade might enhance the in vivo proliferative potential of T3 by increasing the efficiency of CM replication, as may be inferred from the findings of Liu et al.^21^.

In adult mice, the dominant ventricular CM β-AR is the β_1_-subtype^30^. We therefore explored the effects of selective β_1-_AR blockade with metoprolol on T3-stimulated CM proliferation in adults. We found that metoprolol not only increased T3-stimulated ECT2 expression, but it also inhibited basal DUSP5 expression in CMs—a novel function for this receptor. In the presence of metoprolol, short-term T3 therapy robustly built heart muscle in healthy adult mice. Moreover, in chronic post-myocardial infarction (MI) mice with severe progressive heart failure, characterized by extensive pathological left ventricular (LV) remodeling and a marked deterioration of LV systolic function, short-term therapy with metoprolol and T3, given parenterally, regenerated the infarcted LV posterior wall (PW), restoring wall motion and global LV function, as well as effecting reverse remodeling of the heart; salutary effects that persisted for many months after therapy. Given that many heart failure patients already receive β_1_-AR blocker therapy, our findings raise the possibility that a short course of low-dose parenteral T3 therapy, which is also a clinically approved drug, might be sufficient to regenerate postischemic hearts with severe progressive heart failure.

## Results

### β_1_-ARs inhibit T3-stimulated proliferative signaling in CMs

**Figure 1:**
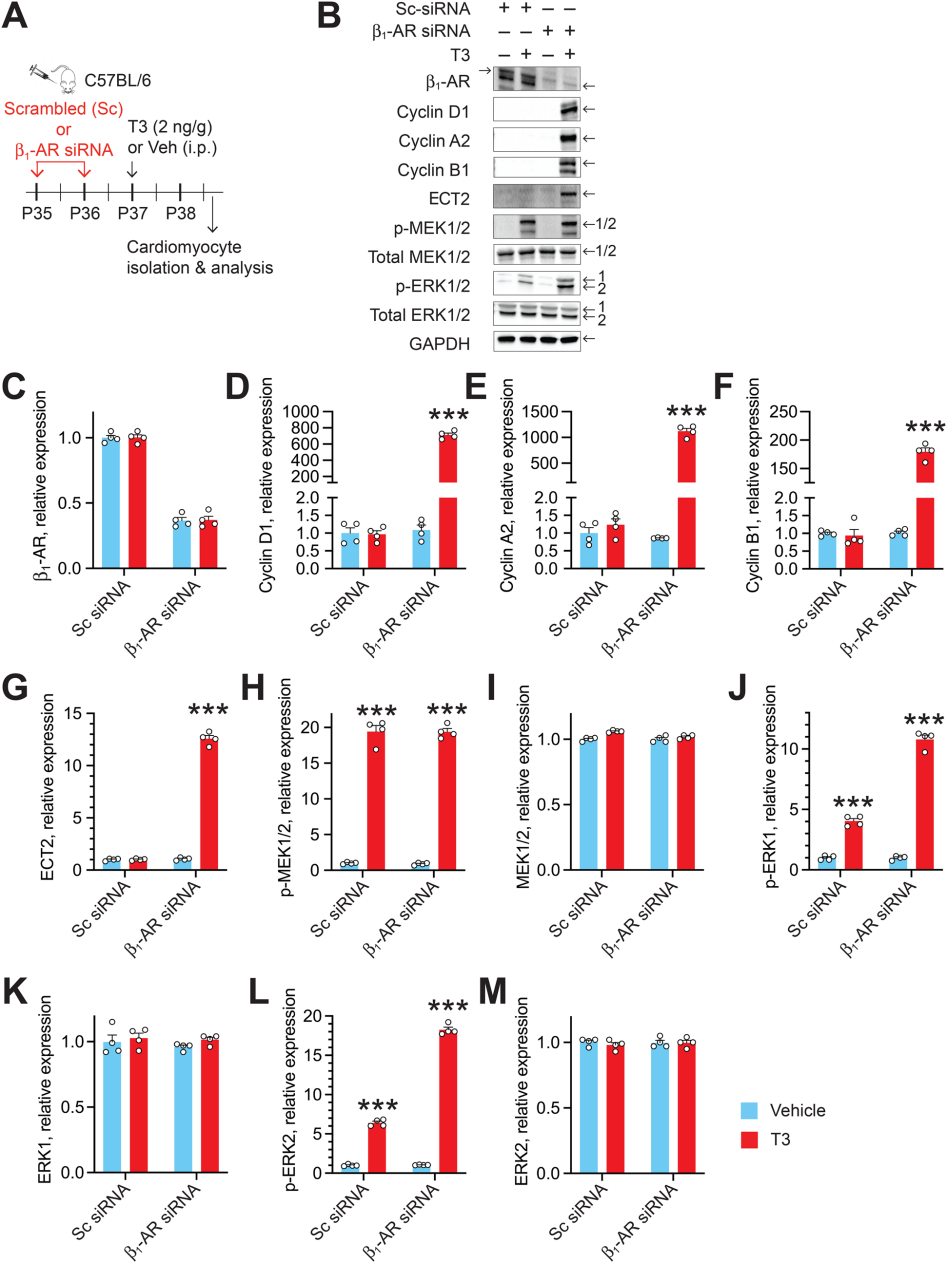
T3-induced ERK1/2 phosphorylation and cyclin expression in adult ventricular CMs is potentiated by β_1_-AR siRNA. (**A**) Schematic showing the protocol for β_1_-AR siRNA and T3 administration and analysis times. (**B**) Representative immunoblots show expression of indicated proteins in lysates obtained from CMs at 40 h after T3 administration. (**C**–**M**) Quantitative data obtained from analysis of immunoblots for β_1_-AR (**C**), cyclin D1 (**D**), cyclin A2 (**E**), cyclin B1 (**F**), ECT2 (**G**), p-MEK1/2 (**H**), MEK1/2 (**I**), p-ERK1 (**J**), ERK1 (**K**), p-ERK2 (**L**) and ERK2 (**M**). The data were normalized with loading control (GAPDH) and data were expressed as relative value for scrambled control siRNA without T3 therapy. Immunoblots are representative of 4 biologically independent replicates. Data are mean ± SEM. ***P < 0.001.

The cell cycle is a series of phases that cells undergo to replicate. Progression through these phases is regulated by cyclin-dependent kinases, which are activated by cyclins. To study the role of β_1_-ARs on the in vivo pro-proliferative action of T3, we used CM-specific expression of cyclins—cyclins D1, A2 and B1 regulate G1/S transition, S phase and G2/M transition—as surrogates for cell cycle activation. We also examined the expression of ECT2, which plays a central role in cytokinesis. For these studies, we administered β_1_-AR siRNA (intraperitoneally, daily over 2 days using the in vivo-jetPEI delivery system) to knockdown β_1_-AR, or non-silencing scrambled siRNA, as a control, to adult mice (Fig. 1A). Then, 40 h after a single intraperitoneally (i.p.) dose of T3 (2 ng/g body weight), we isolated and purified LV CMs. Using immunoblot analysis—representative immunoblots are shown in Fig. 1B—we found that β_1_-AR siRNA decreased CM β_1_-AR expression by 63% (*P* < 0.001) (Fig. 1C). In mice pretreated with β_1_-AR siRNA, but not in mice pretreated with control siRNA, T3 increased cyclins D1, A2 and B1 and ECT2 by ~ 700-fold (Fig. 1D), ~ 1000-fold (Fig. 1E), 190-fold (Fig. 1F), and 12.5-fold (Fig. 1G), respectively, (*P* < 0.001, in each case) (Fig. 1D–G).

T3 increases IGF-1R/MEK1/2-mediated ERK1/2 phosphorylation in CMs^19^. To test whether differential increases in cell cycle promoting proteins in β_1_-AR-containing and -depleted CMs are due to differences in T3-stimulated MEK1/2 and ERK1/2 activation, we determined levels of the phosphorylated forms of these kinases. Phospho-MEK1/2 accumulation after T3 treatment was ~ 20-fold higher (*P* < 0.001), but this increase was not different between β_1_-AR-containing and -depleted CMs (Fig. 1H,I); by contrast, phospho-ERK1/2 levels were ~ threefold higher in β_1_-AR-depleted CMs (*P* < 0.001) (Fig. 1J–M). These findings indicate that, in adult CMs, β_1_-ARs inhibit T3-stimulated p-ERK1/2 accumulation and proliferative signaling; its site of action appears to be distal to T3-stimulated MEK1/2 activation.

### β_1_-ARs promote DUSP5 expression in CMs

T3 stimulation results in a rapid cytoplasmic-to-nuclear translocation of phospho-ERK1/2; its nuclear dephosphorylation in CMs being regulated by DUSP5^19^, a nuclear phosphatase. Because β_1_-AR depletion increases T3-stimulated ERK1/2 phosphorylation without increasing phospho-MEK1/2 (Fig. 1H,I), we hypothesized that β_1_-ARs might sustain DUSP5 expression in CMs. We, therefore, examined the effect of pharmacological β_1_-AR blockade on CM DUSP5 expression. We treated healthy 6-month-old adult mice with metoprolol succinate (metoprolol, 6 µg/g, i.p., daily for 19 days) (Supplementary Fig. S1A), a cardioselective β_1_-AR blocker^31,32^. Ventricular CMs were isolated at multiple points during and after metoprolol therapy and subjected to immunoblotting to detect DUSP5. This analysis showed that DUSP5 expression in CMs was reversibly suppressed by > 98% at 2 weeks after administration of metoprolol (*P* < 0.001) (Supplementary Fig. S1B,C).

To confirm if DUSP5 expression in CMs was specifically regulated by β_1_-AR, we examined the effects of β_1_-AR siRNA or control scrambled siRNA treatment. These siRNAs were administered using the in vivo-jetPEI delivery system^19^. CMs were harvested 64 h after 2 consecutive daily administrations of β_1_-AR siRNA in P35 mice and CMs purified from enzymatically disaggregated LVs using differential centrifugation^19^. This treatment, which reduced CM β_1_-AR expression by 63% (*P* < 0.001) (Fig. 1C), reduced DUSP5 expression by 87% (relative DUSP5 expression in CMs: 1.0 ± 0.028 and 0.13 ± 0.004 in scrambled siRNA and β_1_-AR siRNA treated mice, respectively; *n* = 4 mice/group; *P* < 0.001), indicating that metoprolol-induced inhibition of CM DUSP5 is mediated by β_1_-ARs.

### β_1_-AR blockade promotes T3-stimulated muscularization of healthy LVs

**Figure 2:**
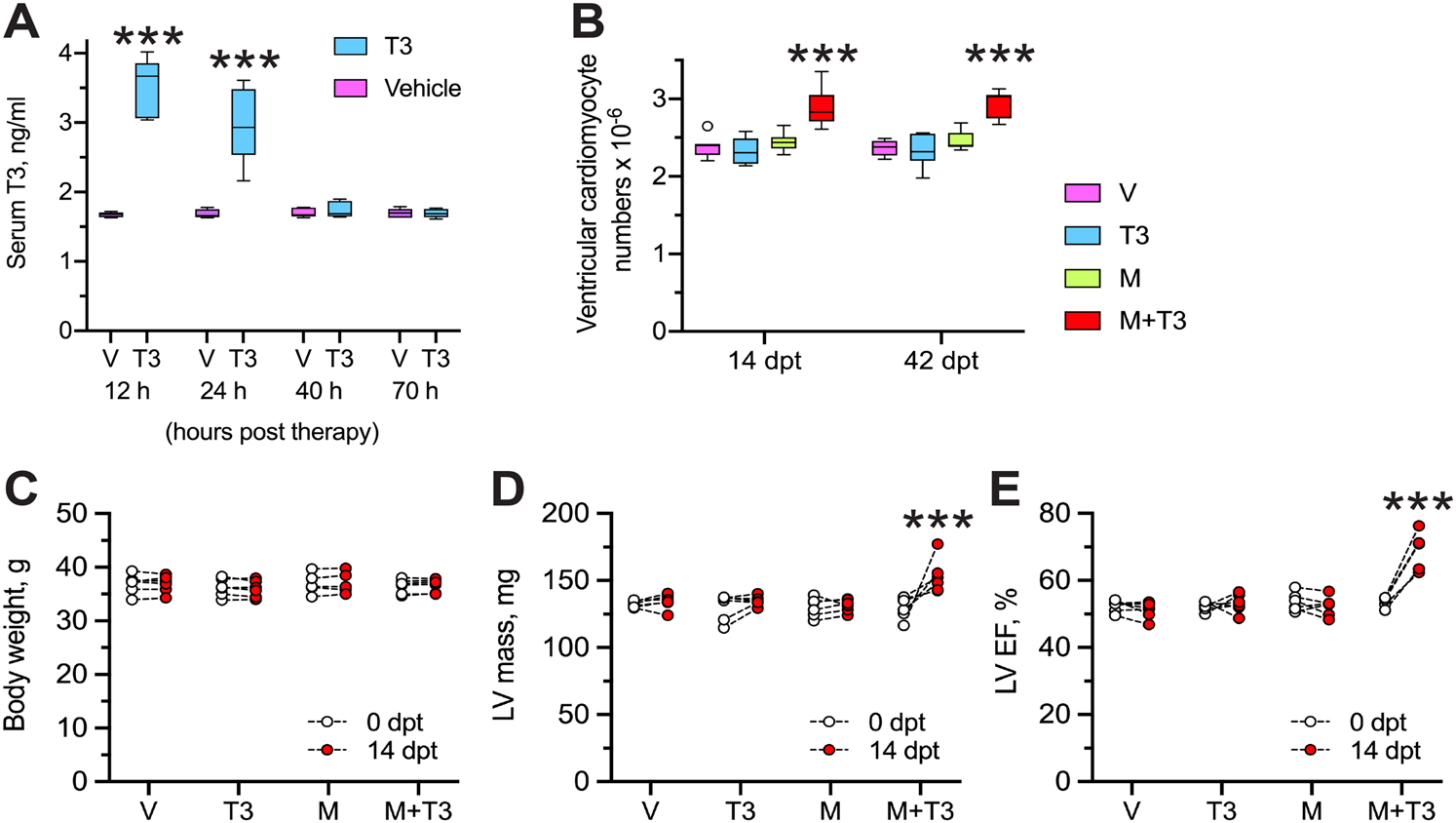
M + T3 therapy muscularizes the adult heart and increases LV contractile function. (**A**) Decay of T3 in the circulation after the end of T3 therapy (2 ng/g body weight, daily for 5 days; i.p.) or vehicle (V) control. Comparisons at each time point were made using an unpaired, 2-sided *t*-test. *n* = 5 mice/group. ***P < 0.001. (**B**) Ventricular CM numbers at 14- and 42-days post-therapy (dpt) with vehicle, T3, metoprolol (M) or M + T3. Comparisons were made using 2-way ANOVA, followed by Sidak’s multiple comparison test. *n* = 7–9 mice/group. ***P < 0.001 for within group (i.e., at 14 or 42 dpt) comparison. (**C**–**E**) Changes in body weight (**C**), LV mass (**D**) and LVEF (E) at 0 and 14 dpt with V, T3, M or M + T3. In each case, comparisons between values at 0 and 14 dpt were made using a paired, 2-sided *t*-test. *n* = 6 mice/group. ***P < 0.001.

To test the in vivo effects of T3, with or without β_1_-AR blockade, on CM proliferation, we administered either metoprolol succinate (6 µg/g, i.p., daily) (hereafter referred to as metoprolol) or vehicle to healthy adult mice for 19 days. T3 (2 ng/g, i.p., daily), or vehicle, was then given concomitantly over the last 5 days, between days 14 and 19 of metoprolol therapy (dual therapy with metoprolol and T3 will hereafter be referred to as M + T3 therapy). We then performed two experiments: (1) to determine the effect of this therapy on circulating T3 levels; and (2) to determine the effects of M + T3 therapy on ventricular CM numbers and LV contractile function. Serum T3 levels were determined on the 5th day of T3 therapy; its decay after cessation of T3 therapy was also followed. This analysis showed that 12 h after the 5th dose of T3, serum T3 levels were ~ twofold higher than in control mice that were given vehicle (*P* < 0.001) (Fig. 2A). Thereafter, serum T3 levels decayed to baseline values over the next 28 h.

To determine the effect of M + T3 therapy on CM proliferation, we sacrificed the mice at either 14- or 42-days post-therapy (dpt). Relative to untreated mice, or those treated with metoprolol or T3 monotherapy, M + T3 therapy increased the total number of ventricular CMs by > 20% (*P* < 0.001) by 14 dpt and this increase was sustained over the next 28 days (Fig. 2B). These data indicate that short-term M + T3 therapy stimulates controlled CM proliferation because its proliferative effect is limited to the immediate post-therapy window.

Since an increase in CM numbers improves LV contractile function^33^, we studied the effect of M + T3 therapy on LV mass and contractile function (ejection fraction (EF)), between the end of therapy and 14 dpt, using M-mode echocardiography. While M + T3 therapy did not change body weight (Fig. 2C), LV mass and EF increased by ~ 18% (*P* < 0.05) and 28% (*P* < 0.0001), respectively (Fig. 2D,E); these parameters were not altered by metoprolol or T3 monotherapy. At 14 dpt, hearts were 13% larger in M + T3-treated mice than in those treated with metoprolol alone (heart-to-body weight ratios were 5.36 ± 0.23 and 4.73 ± 0.05 mg/g in M + T3- and metoprolol-treated mice, respectively; *n* = 6 mice/group; *P* < 0.05), but CMs were 14% smaller (CM areas were 2100 ± 31 and 2400 ± 91 µm^2^ in M + T3- and metoprolol-treated mice, respectively; *n* = 5 mice/group; ~ 200 isolated CMs were sampled from each heart; *P* < 0.01). Thus, M + T3 therapy muscularizes the heart by increasing CM endowment, which increases LV contractility.

### M + T3 therapy reverses severe LV dysfunction in post-MI mice

To evaluate the effects of M + T3 therapy in a model of ischemic cardiomyopathy, we induced a MI in C57Bl/6 mice by permanent coronary artery ligation. C57Bl/6 mice have a relatively low frequency of mononuclear diploid CMs in the naive adult heart and the extent of endogenous cellular regeneration and functional recovery after a MI is relatively low^34^. In these mice, echocardiographic assessments at 30 days post-injury (dpi) revealed LV dysfunction relative to baseline LV function (LVEF ~ 65%), which ranged from modest (LVEF > 50%) to extremely severe (LVEF < 15%). The American College of Cardiology defines severe LV dysfunction as > 30-percentage point decrease in LVEF^5^ (https://www.acc.org/tools-and-practice-support/clinical-toolkits/heart-failure-practice-solutions/left-ventricular-ejection-fraction-lvef-assessment-outpatient-setting).

There is variability in the coronary arterial anatomy of C57BL/6 mice^35^. We selected mice with a 1-month post-MI LVEF between 15 and 30% (that is, a > 35-percentage point decrease from baseline)—prior work suggests that for large infarcts, there is a negative linear relationship between infarct size and LVEF^36^—and then randomly assigned these post-MI mice to treatment groups in a blinded fashion to minimize between-group differences in area of risk after coronary occlusion.

We then conducted a pilot study comparing the effects of M + T3 therapy versus metoprolol monotherapy to determine if the immediate benefit (at 30 days dpt) of therapy was consistent with extensive regenerative repair (which we arbitrarily considered to be a > 70% restoration of pre-MI LVEF) and to determine the sample size for the subsequent study.

Twenty-two mice with LVEFs between 15 and 30% at 1-month post-MI were used for the pilot study (Supplementary Fig. S2A,B); their LV volumes at end-diastole were elevated twofold compared with pre-MI baseline values (50 ± 2 µl and 101 ± 6 µl at baseline and at 30 dpi, respectively; *P* < 0.0001, using a paired *t*-test) indicating that the MI injury resulted in substantial pathological LV remodeling. These mice were then given either metoprolol for 19 days with the addition of T3 over the final 5 days of metoprolol therapy, or only metoprolol monotherapy for 19 days. At 30 days after M + T3 therapy, LVEF increased by 28-percentage points, representing an almost complete reversal of the MI-induced 36-percentage point decrease in LVEF (that is, 78% restoration in LV contractile function) (Supplementary Fig. S2A). By contrast, LVEF did not change significantly after short-term metoprolol monotherapy (Supplementary Fig. S2B). Based on these data, we estimated that a sample size of 4 in each group should have 90% power to show a 25-perentage point increase in post-therapy LVEF at a two-sided significance of 0.01.

**Figure 3:**
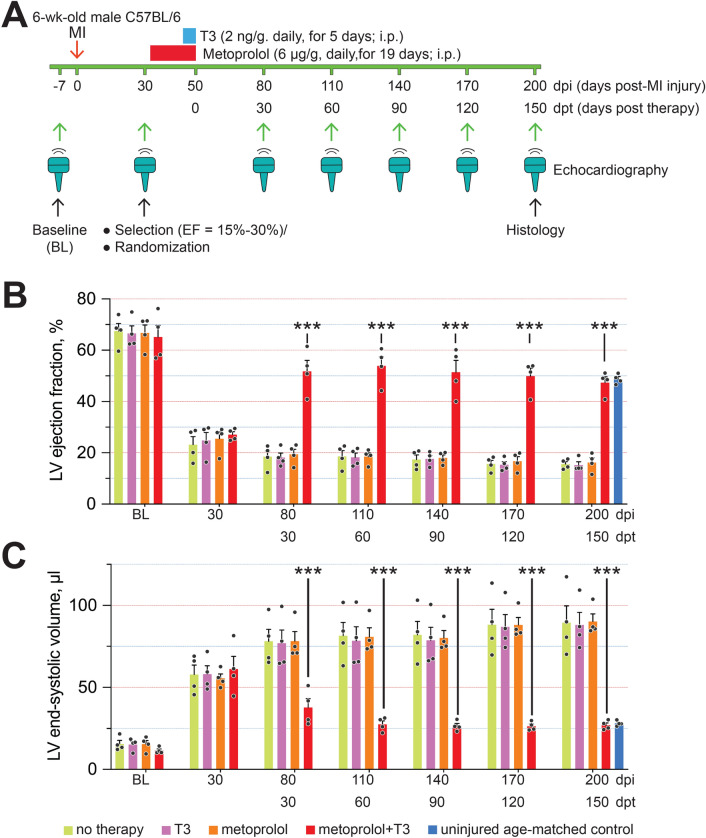
Sustained reversal of systolic heart failure after short-term M + T3 therapy. (**A**) Schematic of the MI experiment in adult mice. The timing and dosage of metoprolol and T3 treatment are indicated (red and blue boxes), as are the timing of MI (red arrow) and duration of posttherapy follow-up. Green arrows indicate when serial echocardiography measurements were taken. (**B**,**C**) LV ejection fraction (**B**) and end-systolic volumes (**C**) of untreated MI-injured mice or those given T3 or metoprolol monotherapy or M + T3 therapy. Data from uninjured age-matched mice are also shown. *n* = 4 mice per group. Individual data points and mean values ± SEM are shown. **P < 0.01, ***P < 0.001 comparing the therapy group to all other post-MI groups at each assessment period.

We then randomized an additional 16 mice with post-MI LVEFs ranging between 15 and 30%, in a blinded fashion, into 4 groups. In addition to a M + T3 therapy group (metoprolol and T3 given as described above), 3 control groups were given either metoprolol for 19 days, T3 monotherapy for 5 days, or no therapy. We studied the initial effects of the treatments at 30 dpt and over the next 120 days (Fig. 3A). M + T3 therapy produced an ~ 30-percentage point increase in LVEF at 30 dpt that persisted throughout the 150 dpt observation period (Fig. 3B); it also rapidly and stably reversed the ~ fourfold post-MI increase in LV end-systolic volume (Fig. 3C). LVEF and end-systolic volume independently predict worsening outcomes in heart failure patients^37^. In contrast to M + T3 therapy, relative to untreated controls, LVEF and end-systolic volume remained unchanged with either metoprolol or T3 monotherapy (Fig. 3B,C) or with no therapy. These data show that M + T3 therapy stably reverses LV systolic dysfunction in mice with severe preexisting post-MI heart failure.

### M + T3 therapy induces renewal by preexisting CMs

**Figure 4:**
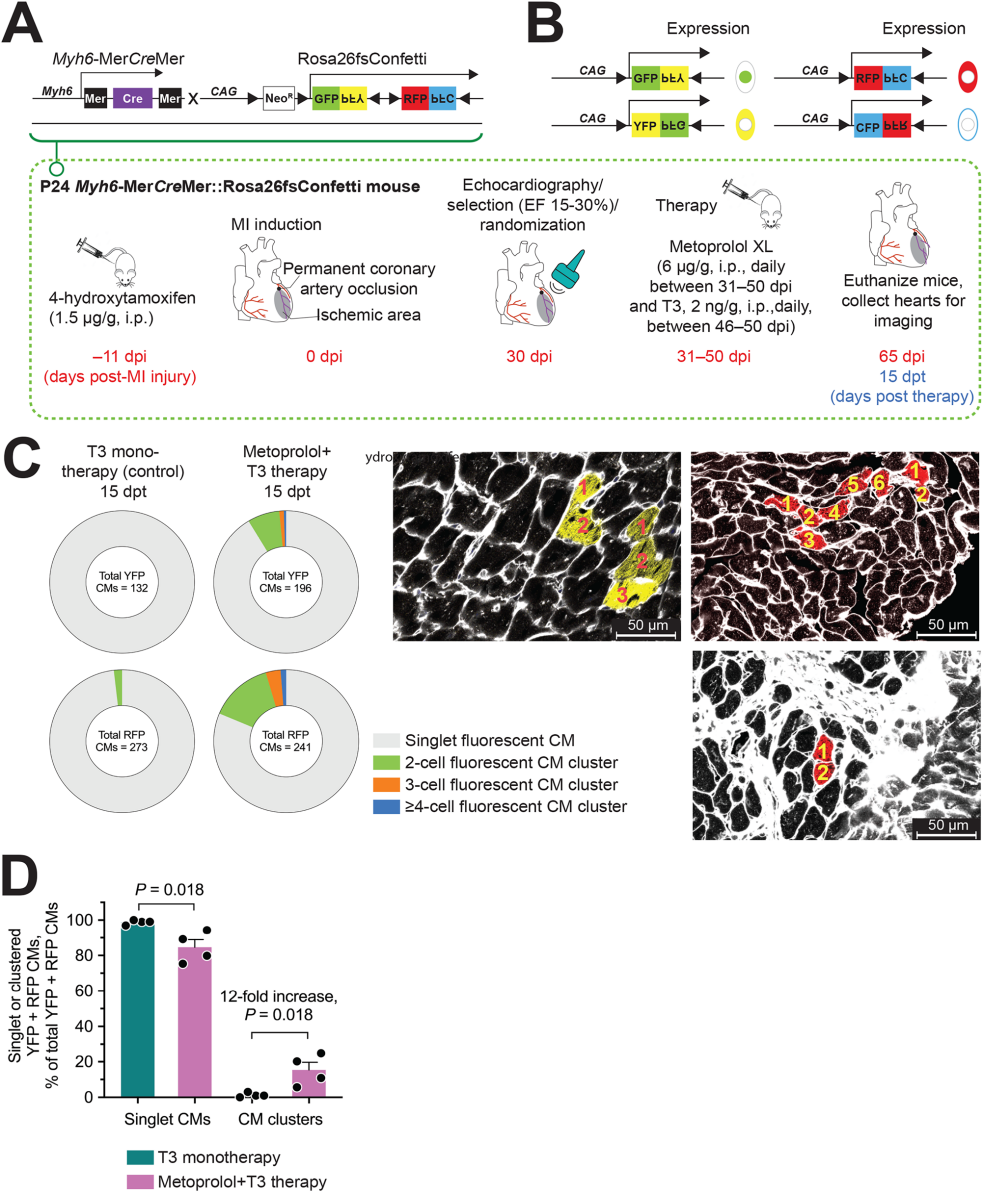
Newly formed CMs in M + T3-treated post-MI hearts are derived from preexisting CMs. (**A**) Schematic showing the Confetti construct and the protocol for pre-MI labeling of CMs by 4-hydroxytamoxifen and subsequent post-MI therapy with M + T3. (**B**) Cre-induced recombination could either induce the expression of green fluorescent protein (GFP), yellow (YFP), red (RFP) or cyan (CFP) in CMs following 4-hydroxytamoxifen administration. (**C**) Pie charts showing RFP- and YFP-expressing monochromatic CM cluster sizes. The number of individual RFP or YFP CMs evaluated for each cluster analysis is indicated in the middle of the Pie charts. Representative images (right) of monochromatic CM clusters with cell outlines delineated using wheat germ agglutinin (WGA)-staining (white). Dense WGA staining (bottom right panel) identifies regions of fibrosis. Numbers indicate contiguous CMs in a cluster. (**D**) Bar graph shows frequency of monochromatic CM clusters at 15 dpt. *n* = 4 mice per group. Individual data points and mean values ± SEM are shown.

We then tested the hypothesis that, in hearts with severe ischemic injury, M + T3 therapy stimulates CM renewal from preexisting CMs. We sought evidence of such renewal using multicolor lineage tracing in double-transgenic *Myh6*-Mer*Cre*Mer::Rosa26fs-Confetti mice^38^, an approach that allows the simultaneous evaluation of the proliferative trajectories of many LV CMs (Fig. 4A). In these mice, Cre recombinase activation upon 4-hydroxytamoxifen (4-HT) administration causes the Confetti construct to recombine, which randomly labels CMs with red fluorescent protein (RFP), yellow fluorescent protein (YFP), green fluorescent protein (GFP) or cyan fluorescent protein (CFP) (Fig. 4B). The administration of 4-HT at a previously determined dose (1.5 µg/g, i.p.)^26^ to these mice causes recombination-driven RFP and YFP expression in CMs, as determined by confocal microscopy, at frequencies that minimize the occurrence of monochromatic CM clusters^26^, a prerequisite for multicolor analysis of discrete CM replication events. However, at this low 4-HT dose, GFP- and CFP-positive CMs are not observed^26^. Eleven days after we gave 4-HT to *Myh6*-Mer*Cre*Mer::Rosa26fs-Confetti mice, we subjected them to MI and, upon confirmation of injury at 30 dpi (LVEF < 30% but > 15%), we administered M + T3 therapy or, as a control, T3 monotherapy, as before, and imaged the hearts at 15 dpt (Fig. 4A). Because T3 increases transcription at the *Mhy6* promoter, it has the potential to increase *Cre* expression. To obviate this confounding effect, we used T3 monotherapy as a control for M + T3 therapy. M + T3 therapy resulted in a 12-fold increase (*P* = 0.018) in monochromatic CM clusters (Fig. 4C,D); the most frequently observed monochromatic CM clusters contained 2 CMs (Fig. 4C), indicating cell replication. A few clusters had 3 consecutively-labeled cells and fewer still had 4 to 6 consecutively-labeled cells, which signified multiple rounds of CM replication. These findings provide evidence for the involvement of preexisting CMs in M + T3-induced remuscularization.

**Figure 5:**
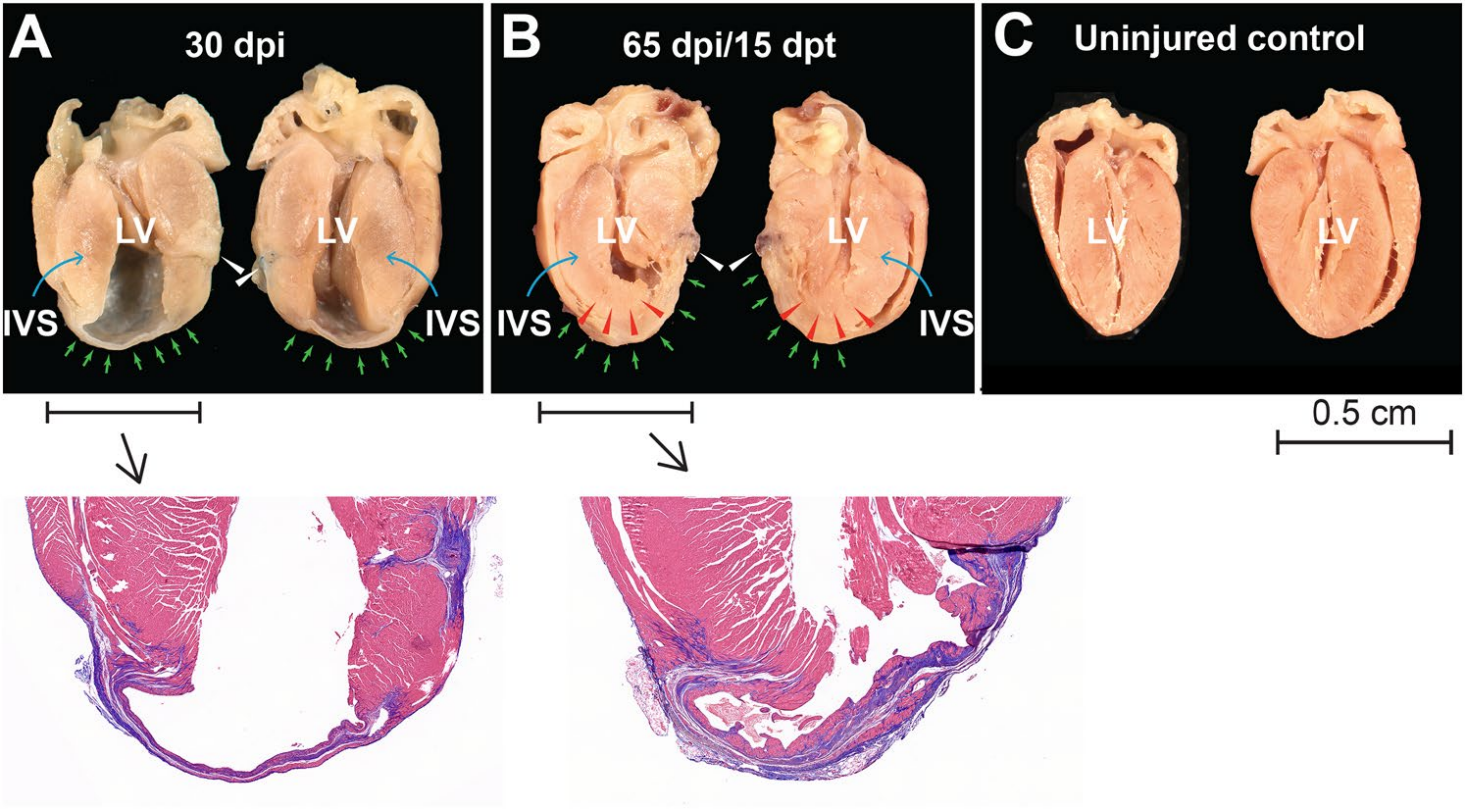
Early regrowth of the post-infarcted ventricular myocardium after M + T3 therapy. (**A**,**B**) Longitudinally cut hearts (both halves) of a 30 days post-MI injury (dpi) (**A**) and a 15 days post-therapy (dpt) (**B**) heart. The white arrowheads show the site of coronary artery ligation, the green arrows show the infarct and the red arrowheads show extension of the intraventricular septum (IVS) toward the ligature site. Insets show trichrome-stained sections from indicated hearts. (**C**) An uninjured age-matched control heart. Each image set is representative of 2–4 hearts at each time point.

To visualize the near-term morphological consequences of M + T3-stimulated CM proliferation, we examined gross anatomy and trichrome-stained tissue sections of post-MI LVs before therapy and then at 15 dpt; scar tissue being shown in blue and viable myocardium in red (Fig. 5A,B). Before therapy (at 30 dpi), a thin scar was evident between the LV apex and the mid-apical PW (halfway between the apex and the mid-papillary muscle) (Fig. 5A, green arrows). At 15 dpt, new myocardium appeared to extend from the apical tip of the intraventricular septum (IVS) toward the LV mid-apical PW (Fig. 5B, red arrowheads). The gross anatomy of an uninjured age-matched control is shown for comparison (Fig. 5C). Together, these data indicate the rapidity of regenerative repair, which involves remuscularization of the scarred LV, and is evident as early as 15 dpt.

### Long-term consequences of M + T3 therapy on the LV structure and function

**Figure 6:**
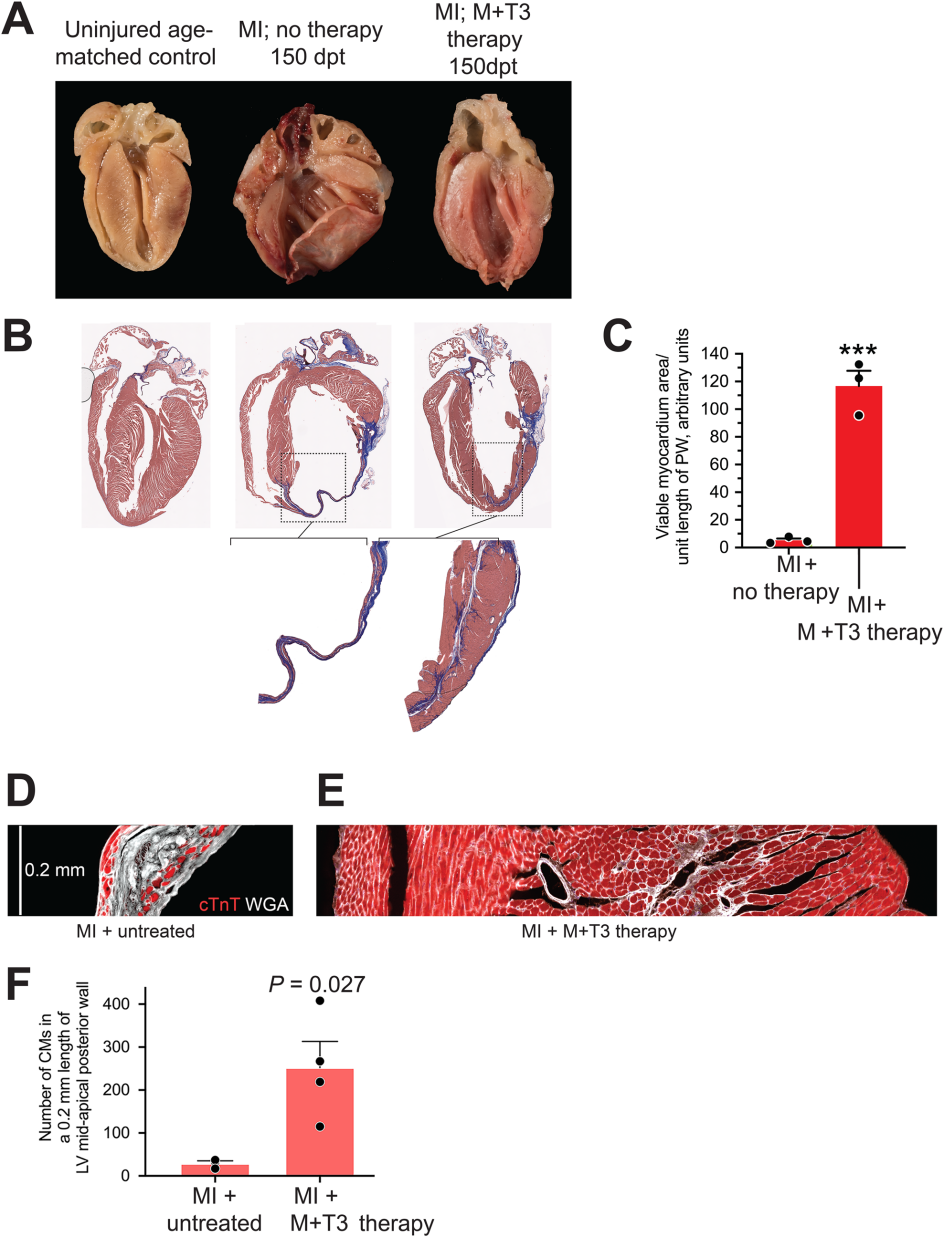
M + T3 therapy remuscularizes the infarcted mid-apical LV PW. (**A**,**B**) Gross anatomy (**A**) and trichrome staining (**B**) of hearts from mice that were either uninjured (left), left untreated after an MI (middle) or treated with M + T3 therapy post-MI (right) at 150 days post-therapy (dpt). The images are representative of 3–4 hearts per group. The insets in (**B**) show a magnified view of the boxed region. Supplementary Fig. S4A–E, show other examples of gross anatomy of post-MI heart, including those shown in “A”, that were untreated or given T3 monotherapy, metoprolol monotherapy or M + T3 therapy. (**C**) Quantitative analysis of viable myocardium in the LV mid-apical PW at 150 dpt. Data represent values from 3 independent hearts per group. ***P < 0.001. (**D**,**E**) Representative longitudinal tissue section (7 µm thick) images, representing 0.2 mm-long views of the LV posterior wall at the mid-apical region, from an untreated post-MI mouse (**A**) and from a post-MI mouse after M + T3 therapy (**B**). These hearts are from mice followed for 150 dpt. CMs are visualized using cardiac troponin T (cTnT) staining (red) and cell boundaries are outlined by wheat-germ agglutinin (WGA) staining (white). WGA-staining also indicates areas of scar tissue and fibrosis. In these longitudinal images of the LV posterior wall, the LV cavity is on the left side of the tissue section. (**F**) Number of CMs (CMs) in 0.2 mm-long tissue section views of the LV mid-apical wall from post-MI untreated and post-MI M + T3-treated hearts. Data represent values from 3–4 independent hearts per group. An unpaired 2-sided student’s t-test was used to compare differences between groups. Data are shown as individual values and mean ± SEM.

Heart failure is a progressive disease that is characterized by cellular (for example, CM hypertrophy), as well as regional and global structural and functional changes resulting from a loss of viable myocardium and increased wall stress^39–41^. We, therefore, addressed the ability of M + T3 therapy to reverse some of these processes in hearts with severe preexisting ischemic injury. At 150 dpt, assessments of gross LV morphology and visualization of fibrosis and viable myocardium (using trichrome staining) indicated the formation of a new myocardium surrounding a fibrous core (Fig. 6A, right and Fig. 6B, right), which is a remnant of the scarred LV PW observed at 30 dpi (Fig. 5A). Quantitative measurements, at 150 days after M + T3 therapy, indicated that the amount of viable myocardium per unit length of the LV mid-apical PW was > 20-fold higher than in untreated mice (*P* < 0.001) (Fig. 6C). Using immunohistochemical markers of CMs (cardiac troponin T, cTnT) and cell wall (wheat germ agglutinin, WGA), we show that the remuscularized myocardium of M + T3 therapy-treated post-MI mice have ~ tenfold greater number of CMs than those of untreated mice (*P* = 0.027) (Fig. 6D–F). To examine the extent of angiogenesis, we compared the capillary densities of the remuscularized LV PW with those of uninjured hearts, using high power microscopy. We found that the capillary-to-CM ratio was similar between these mouse groups (Supplementary Fig. S3), suggesting that the process of rebuilding heart muscle increases angiogenesis to match CM proliferation. We also determined collagen deposition (blue area in trichrome stained sections) (Fig. 6B) in LV mid-apical PW segments, which showed that M + T3 decreased scar area (by 64%), but this change was not statistically significant (relative collagen areas/unit PW length were: 1.0 ± 0.3 and 0.36 ± 0.06 in 150 dpt vehicle and M + T3 therapy groups, respectively; n = 3/group; *P* = 0.11).

**Figure 7:**
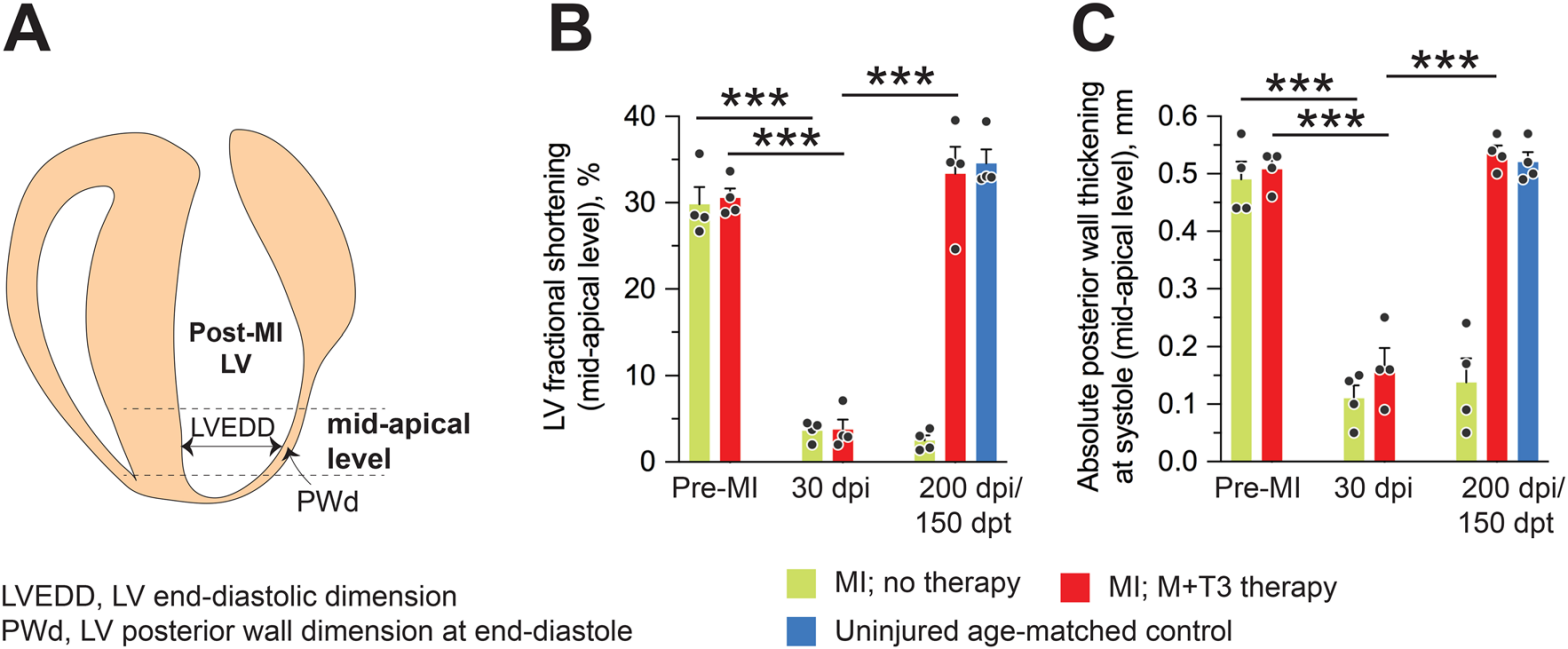
M + T3 therapy restores motion to a post-infraction segment of LV mid-apical PW. (**A**) Schematic of a post-MI heart showing the site of echocardiographic measurements. (**B, C**) Quantitative analysis of LV mid-apical fractional shortening ((LV end-diastolic dimension − end-systolic dimension) × 100/LV end diastolic dimension) (**B**) and absolute PW thickening (mid-apical LV PW thickness at end-systole – end-diastole) (**C**) at 30 dpi and 150 dpt. *n* = 4 mice per group. ***P < 0.001. Individual data points and mean values ± SEM are shown. See also Supplementary Movies S1–S3.

The pump function of the LV is compromised progressively by a MI, as was evident by reductions in LVEF (Fig. 3B). This LV dysfunction mostly results from a loss of PW motion between the papilla and the apex. As the LV PW contracts, the LV chamber dimension decreases and its wall thickens. At 30 dpi, fractional shortening and end-systolic PW thickening at the mid-point between the papilla and the apex decreased by ~ 90% and ~ 70%, respectively (*P* < 0.001, in each case) (Fig. 7A–C). In contrast, 150 days after acute M + T3 therapy, these variables were similar to those of uninjured hearts (Fig. 7A–C). Sequential B-mode images (parasternal long axis views) of a representative mouse heart demonstrate the debilitating effect of MI-injury on LV PW motion (Supplementary Videos S1, S2), and then the transformation of a mostly akinetic LV mid-apical PW at 30 dpi to one in which PW motion had been reestablished at 150 dpt (Supplementary Videos S2, S3).

**Figure 8:**
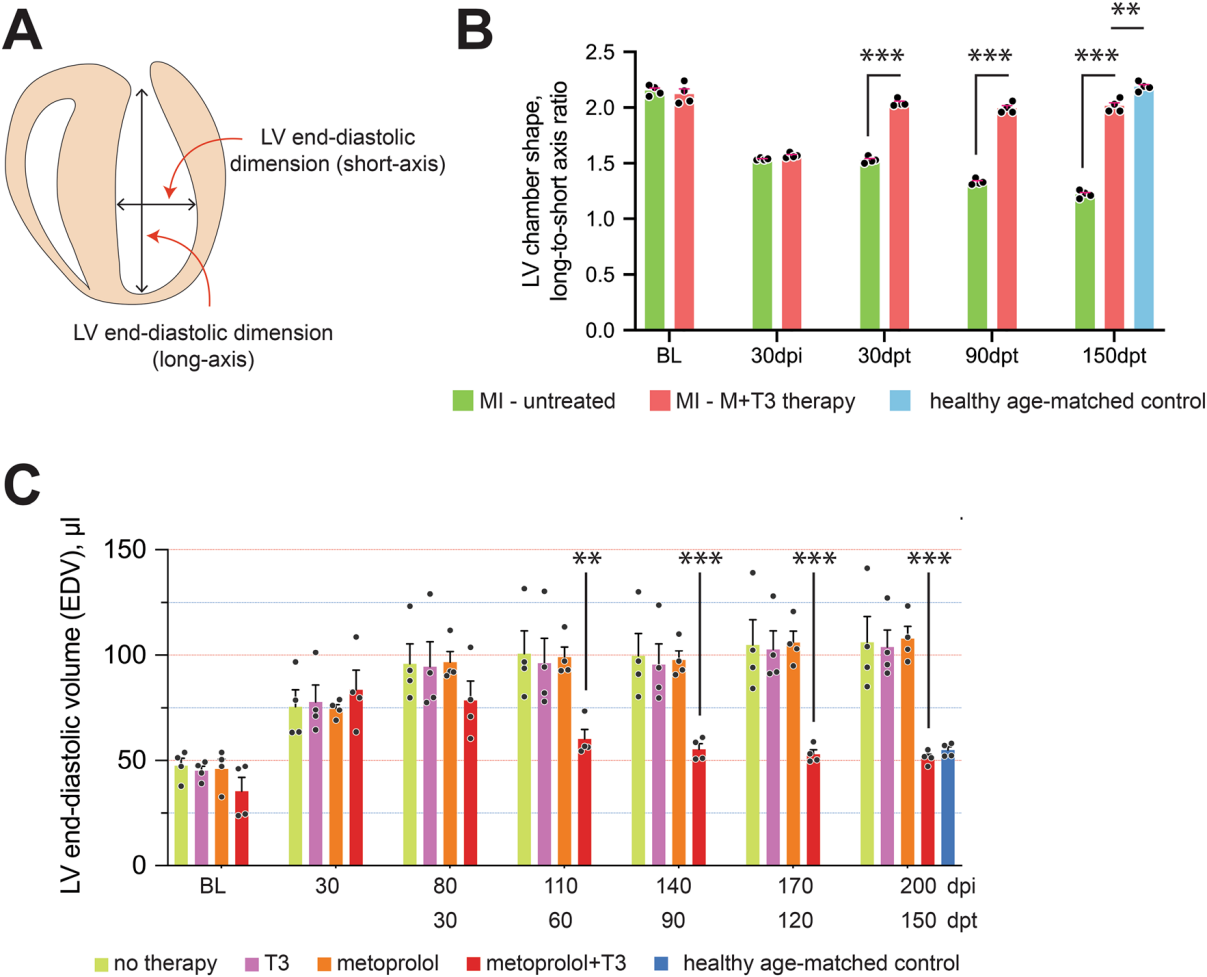
M + T3 therapy reverse-remodels the post-MI LV. (**A**) Illustration showing the LV end-diastolic long- and short-axis dimensions, which were determined using echocardiography. (**B**) Bar graph showing long-to-short axis ratio before MI surgery (baseline, BL), at 30-days post-MI injury (30 dpi) and at 30-, 90- and 150-days after cessation of M + T3 therapy (days post-therapy, dpt). *n* = 4 mice per group. Data are shown as individual values and the mean ± SEM. Comparisons were made by two-way ANOVA followed by Sidak's multiple comparisons test. **P < 0.01, ***P < 0.001. (**C**) LV volumes at end-diastole of untreated MI-injured mice or those given T3 or metoprolol monotherapy or M + T3 therapy. Data from uninjured age-matched control hearts are also shown. Two-way analysis of variance (ANOVA), followed by Sidak's multiple comparisons test. **P < 0.01, ***P < 0.001 comparing the combination therapy group to all other post-MI groups at each assessment period. *n* = 4 mice per group. Data are shown as individual values and the mean ± SEM.

Severe heart failure is also characterized by altered LV geometry and chamber dilatation (increased end-diastolic volume). At 30 dpi, LV geometry changed from that of a prolate ellipsoid to being more spherical (Fig. 8A,B), and LV end-diastolic volume increased by > twofold (*P* < 0.001) (Fig. 8C). In marked contrast, by 150 dpt, the LV chamber was much less dilated (Fig. 8C) and more conical in shape (Figs. 6A and 8B). Thus, M + T3 therapy produced an enduring reversal of MI-induced defects in LV structure.

CM hypertrophy in the spared LV myocardium (that is, the remote zone) is a hallmark of post-MI remodeling. We looked for evidence of CM hypertrophy in the LV remote zone as well as in the remuscularized LV mid-apical PW (Supplementary Fig. S5A). Estimation of CM cross-sectional areas revealed that LV remote zone CMs of M + T3-treated post-MI hearts were ~ 30% smaller than those of untreated post-MI hearts (*P* < 0.001), but similar in size to those of uninjured hearts (Supplementary Fig. S5B). We also found that the mean cross-sectional area of LV mid-apical PW CMs of M + T3 treated post-MI mice was similar to that of uninjured mice (Supplementary Fig. S5C). Collectively, these findings indicate that M + T3 therapy stably rebuilds the myocardium where previously there was an injury and, in uninjured LV regions, it prevents both CM hypertrophy and increases in LV end-diastolic volume, the hallmarks of pathological remodeling.

## Discussion

The key objective of regenerative therapy is to redress severe postischemic heart failure by restoring lost LV myocardium and, thereby, improving contractile function. A more intractable objective is restoration of organ architecture which, in the case of chronic ischemic cardiomyopathy, contributes to increased LV wall stress and results in progressive deterioration of LV function. Another objective is to develop therapies that have high translational potential. Here, we describe the development and use of a therapeutic regimen that fulfils these objectives, which heretofore has remained elusive.

In terms of gross morphology, LV muscularization is an increase in LV myocardial tissue volume or LV mass. But, at the cellular level, LV muscularization can occur in distinct ways: through hypertrophy, hyperplasia or both. Stressors, such as pressure overload, cause CMs to hypertrophy, which is initially compensatory but, long term, such hearts frequently progress to failure^41,42^. In LVs in which CMs can be forced to proliferate without cellular hypertrophy muscularization increases LV contractility^26^. However, excessive stimulation of CM proliferation can be detrimental. For example, overexpression of a constitutively active mutant of ERBB2 not only results in profound CM proliferation, but also dedifferentiation and hypertrophy and, ultimately, increased mortality^17^.

Here we show that M + T3 therapy, given transiently to healthy adult mice, increases ventricular CM endowment. We demonstrate this increase both indirectly and directly. We found that, 14 days after the cessation of M + T3 therapy, LV mass was 18% higher at a time when LV myofiber mean cross-sectional area was 14% lower than that of controls. The most parsimonious explanation of this finding is that the LV myocardium of post-M + T3 hearts has more CMs. The decrease in CM size not only means that M + T3 therapy is not hypertrophy-inducing, but results in cell replication because daughter cells are initially smaller than their parent cells. To confirm this conclusion, we directly counted ventricular CMs after enzymatic disaggregation of the heart, which revealed a 20% increase in CM numbers. Importantly, this increase, which was sustained over the following 28 days, cannot be attributed to differences in CM number estimation between M + T3 therapy and control groups resulting from incomplete enzymatic digestion of the myocardium, since we carefully quantitated the efficiency of tissue disaggregation and found it to be over 97% in both control and treated animals.

In healthy adult mice, M + T3 therapy increased LVEF by a remarkable 28% and did not result in any death in the 42 days of post-therapy follow up. Thus, the finding of an increase in CM endowment coupled with an increase in LV mass and contractile function is consistent with M + T3 therapy inducing therapeutic LV muscularization.

The mechanistic basis for M + T3-induced therapeutic muscularization is worthy of consideration. A working model is presented in Supplementary Fig. S6. T3 increases IGF1 and IGF-1R transcription^18^, which activates ERK1/2^18^. ERK1/2 signaling has diverse cellular functions: while cytoplasmic retention of phosho-ERK1/2 stimulates CM hypertrophy, a sustained increase in nuclear phospho-ERK1/2 stimulates cell cycle activity^17,43^. T3/IGF-1/IGF-1R signaling causes rapid nuclear translocation of phospho-ERK1/2 in P8 CMs^19^. Despite this effect, meaningful CM proliferation does not occur unless in vivo T3 dosing is continued over several days (~ 3–5 days)^18,19^. These effects of exogenous T3, specifically mediated through CM IGF-1R activation, are key to its proliferative action^18^. However, as CMs mature, a developmental increase in DUSP5, a nuclear phospho-ERK1/2-specific phosphatase, counteracts both T3-stimulated accumulation of phospho-ERK1/2 in the nucleus and CM proliferation^19,26^.

We show here that β_1_-AR blockade and T3 therapy increases CM numbers in adult mice. The key finding that β_1_-ARs negatively regulate T3-stimulated phospho-ERK1/2 buildup in CMs, but not the accumulation of its upstream activator MEK1/2, implicates phospho-ERK1/2 dephosphorylation by its specific phosphatase DUSP5 as a β_1_-AR target. Consistent with this notion, we found that β_1_-AR depletion (using siRNA-mediated receptor knockdown) inhibited CM DUSP5 expression and increased T3-stimulated phospho-ERK1/2 in CMs. Also, β_1_-AR blockade reversibly inhibited DUSP5 expression in CMs and synergized with T3 to increase CM endowment. The effects of β_1_-AR blockade were identical to those observed with genetic or pharmacological inhibition of DUSP5, which is necessary and sufficient for suppressing T3-induced CM proliferation and therapeutic LV muscularization^19,26^.

CM dedifferentiation frequently^44^, but not always^8,18^, accompanies CM proliferation; in cases where dedifferentiation occurs, LV function is depressed^9^. In murine CMs, we found that T3 not only increases the expression of multiple cell cycle-promoting genes, it also increases expression of *Mhy6* and *Tnni1*, key markers of CM differentiation, as well as genes that control oxidative phosphorylation^18^, again markers of mature CMs. These effects, which result from direct genomic actions of T3^24^, may explain why the CM regeneration observed with M + T3 therapy is not associated with a decrease in LV function (Fig. 2E).

Injury-induced cardiac regeneration is observed in adult zebrafish and neonatal mice^16,45^. CMs of these hearts have high proliferative potential that is activated by the injury. But the molecular and cellular signals caused by injury must be short lived because CMs become quiescent after repair is complete. Repairing hearts long after the injury—that is, those with chronic preexisting disease—requires a remuscularization stimulus that is independent of that produced by injury. Because of its effectiveness in enhancing muscularization in healthy animals, we examined the potential for M + T3 therapy to repair hearts long after MI.

We studied therapeutic remuscularization with short-term M + T3 therapy only in hearts with severe injury. We found that short-term M + T3 therapy remuscularizes the thin heavily scarred post-MI LV PW and that its effects are enduring. At 150 dpt, mid-apical viable myocardial area was more than 20-fold higher in M + T3 treated mice than in untreated controls. Histological analysis indicated that these thicker LV PW segments contained ~ tenfold more CMs per unit wall length than those of untreated post-MI hearts (Fig. 6D–F). Because wall stress is inversely proportional to wall thickness, therapeutic remuscularization is expected to reduce regional wall stress, which is an independent predictor of LV remodeling after MI^46^. By 150 dpt, M + T3 therapy had effectively reverse remodeled the heart leading to a normalization of overall LV shape (Fig. 8) and regression of CM hypertrophy (Supplementary Fig. S5). Lack of LV PW motion, or akinesis, is a serious complication of MI. Serial assessments of end-systolic and end-diastolic LV cavity dimensions at the mid-apical level of each heart, between 30 dpi and 150 days post-M + T3 therapy, showed that M + T3 therapy caused a > sixfold increase in regional fractional shortening. Thus, we show concordance between direct (increased viable myocardium in the scarred LV PW and increased regional LV fractional shortening) and indirect (reversal of remote zone CM hypertrophy and reverse-remodeling of the LV chamber) effects of M + T3-induced remuscularization.

The use of a cardioselective β_1_-AR blocker, together with T3, to remuscularize MI-injured LVs would suggest that preexisting CMs are a source of new CMs; CMs abundantly express β_1_-ARs^30^. We sought evidence to support this contention using lineage tracing, the technique of choice to identify progeny of single cells^47^. With these studies, specificity is provided by the use of a *Myh6* promoter to drive tamoxifen-dependent Cre recombinase expression, which results in monochromatic fluorescent labeling of CMs with a fluorescent protein. Moreover, by using low-dose 4-HT, random occurrence of monochromatic CM clusters is minimized. The additional use of WGA labeling, which identifies cell borders in tissue sections, distinguishes one CM from another.

Using *Myh6*-Mer*Cre*Mer::Rosa26fs-Confetti mice, we found that M + T3 therapy increases the frequency of LV monochromatic CM clusters by 12-fold. Such clusters, which are mostly non-existent after control therapy, signify the conversion of labeled CMs into adjacently-positioned, similarly labeled daughters; that is, replication of preexisting CMs, which was observed in all post-MI LV regions. Our finding that β_1_-AR depletion potentiates T3-stimulated expression of ECT2 in CMs (by > 12-fold)—ECT2 facilitates hepatocyte cytokinesis during liver regeneration^48^—could explain the efficacy of M + T3 therapy in stimulating CM replication.

MI induces a surge in CM death over the first 6–24 h of ischemic injury^49^. T3 therapy, instituted at the time of ischemic injury, has been shown to stimulate cardioprotective prosurvival pathways^50^. We do not believe, however, that this action of T3 has an important role in the beneficial effects of M + T3 regenerative therapy in the setting of preexisting heart failure, because in our studies M + T3 therapy was initiated one month after MI, where levels of CM apoptosis are negligible^49^. It is perhaps for this reason that the marked increase in viable myocardium in the mid-apical LV PW was associated with a much smaller (and non-significant) decrease in scar area. However, it will be important to evaluate if late cardiac remuscularization therapy with M + T3 can be complimented with early T3 monotherapy to limit scar formation ab initio.

Effective cardiomyogenesis—that is, the process leading to the formation of myocardium—also involves neovascularization, reinnervation and restoration of the extracellular matrix^51^. It will be important to study the effect of M + T3 therapy on these processes and to determine their role in the regenerative response observed with this therapy. Here, we examined capillaries as a part of the cardiac regenerative response and found that the CM-to-capillary ratio was near normal after M + T3 therapy. This suggests that, post-M + T3 therapy, neoangiogenesis was commensurate with LV remuscularization. This may not be surprising, given that CMs elaborate angiogenic factors, such as vascular endothelial growth factor^52^.

In this era when percutaneous coronary interventions being widely used as primary therapy for occlusive myocardial disease, myocardial damage is now more commonly due to ischemia–reperfusion injury than to permanent coronary artery occlusion injury^53^. However, even with the widespread use of primary percutaneous intervention, ~ 15–25% patients are not successfully reperfused in a timely manner^40^. The key objective of our study was to determine the effect of remuscularization in hearts with severe LV dysfunction and extensive LV remodeling. Long-term remodeling is more robustly seen after MI due to permanent coronary artery occlusion, reducing the sample size needed to detect differences between groups^40^. We note that M + T3 therapy did not repair the LV PW immediately distal to the occlusion. It will be important to determine if ischemia–reperfusion injury is more completely repaired with M + T3 therapy than permanent coronary occlusion injury.

In summary, using a murine model of ischemic cardiomyopathy with marked cardiac remodeling and severely impaired LV function, we show that short-term T3 therapy together with β_1_-AR blockade remuscularizes the heavily scarred LV PW, which results in an enduring improvement in LV structure and contractile function.

### Potential for translation

In the United States, the long-acting β_1_-AR blocker metoprolol succinate is specifically approved for the treatment of heart failure due to structural heart disease^54^. Thus, in many heart failure patients on metoprolol therapy, future regenerative therapy might simply involve the addition of short-duration T3 therapy. A recent Phase II randomized clinical trial has examined the effect of long-term (6-month) T3 therapy in patients with a prior MI and low-T3 syndrome^55^. While the therapy was considered safe and reduced regional contractile dysfunction, subgroup analysis of patients receiving β_1_-AR blocker therapy was not performed. It is difficult to extrapolate findings from this clinical trial to the translational value of M + T3 therapy because the investigators of this clinical trial used T3 therapy to reverse the low-T3 syndrome, but not to elevate circulating T3 levels beyond the normal range which, in mice, is necessary for inducing CM proliferation. Given the findings here, short-duration T3 therapy, at doses that elevate circulating T3 beyond the normal range in heart failure patients receiving metoprolol therapy, may prove useful in permanently reversing LV dysfunction and therefore warrants further clinical investigation.

## Materials and methods

A detailed description of the experimental procedures related to cardiomyocyte isolation for number determination, immunocytochemical studies with antibodies, histology and immunoblotting is provided in the Supplementary Information section.

### Animal care and use

Mice were housed under pathogen-free conditions in a facility approved by the American Association for the Accreditation of Laboratory Animal Care. All animal studies were approved by the Institutional Animal Care and Use Committee (IACUC) of Emory University. We confirm that all experiments were performed in accordance with IACUC guidelines and regulations. We also confirm that the study is reported in accordance with ARRIVE guidelines. C57Bl/6 wild type (Jackson Laboratory, 000664) male mice were used for the studies reported here. We performed cardiomyocyte (CM) lineage tracing studies as previously described^19,26^ (21, 28) using Rosa26fs-Confetti [B6.129P2-Gt(ROSA)26Sortm1(CAG-Brainbow2.1)Cle/J, Jackson Laboratory, 017492] and *Myh6*-MerCreMer [B6.FVB(129)-A1cfTg(Myh6-cre/Esr1*)1Jmk/J, Jackson Laboratory, 005657] mice which were crossbred to generate double-transgenic *Myh6*-MerCreMer::Rosa26fs-Confetti mice. The Rosa26fs-Confetti construct is also termed, R26R-Confetti^38^. As previously detailed^26^, Cre-mediated recombination was optimized by adjusting the dose of 4-hydroxytamoxifen (4-HT) (H7904-5 mg, Sigma) to minimize replication-independent occurrences of adjacent CMs of the same color. All animals belonged to the C57Bl/6 strain, were healthy, immune-free, and drug or test naive and were not involved in other experimental procedures. Littermates were used as controls for all lineage tracing experiments.

### Administration of drugs

T3 (Sigma-Aldrich, T6397-100MG), metoprolol (metoprolol succinate, 200 mg; catalog number 1441298) and 4-HT were administered, intraperitoneally (i.p.). Our prior studies^19^ show that 5 doses of T3 (2 ng/g, daily, for 5 days; i.p.) sustain ERK1/2 activation in CMs, which is necessary for in vivo CM proliferation; fewer doses of T3 did not consistently or robustly stimulate CM proliferation when given in combination with DUSP5 depletion in CMs (data not shown). Moreover, while i.p. T3 administration (2 ng/g) increased circulating T3 levels, the extent of this increase differed between operators. For this reason all drug administrations were performed by one investigator (L. T.); in these studies 2 ng/g T3 increased circulating T3 levels by about twofold (e.g., Fig. 2A). Metoprolol was injected daily (6 µg/g body weight) for 14 days before the start of T3 and then continued for another 5 days in combination with T3. The rationale for this metoprolol dosing protocol is based on our studies showing nearly complete suppression of DUSP5 expression in CMs using this protocol (Supplementary Fig. S1); higher doses of metoprolol have also been used to selectively block the β_1_-AR^56^. Genetic lineage tracing studies used *Myh6*-MerCreMer::Rosa26fs-Confetti mice. A single dose of 4-HT was administered (1.5 µg/g body weight, i.p.) 41 days before the start of M + T3 therapy. This dose, which was based on dose titration studies^26^, minimizes replication-independent occurrence of monochromatic clusters by ensuring minimal labeling of CMs (~ 1% of the heart)^26^.

β_1_-AR-specific siRNA (sc-29581, Santa Cruz Biotechnology) or scrambled siRNA (control) was administered using in vivo-jetPEI, (VWR, 89,129–960). β_1_-AR siRNA (100 ng) was dissolved in 1 ml of the in vivo-jetPEI:10% glucose mixture and was injected 100 μl per mouse via i.p. route (10 ng/mouse). β_1_-AR siRNA was a pool of 2 different siRNA duplexes.

### Murine model of ischemic cardiac injury

MI injury was induced by permanent ligation of the left anterior descending artery. Surgeries were performed under aseptic techniques. Body temperature was maintained using warm-water blankets. Under aseptic conditions and anesthesia, mice were orally intubated using polyethylene-60 (PE-60) tubing. The tubing was connected to a rodent ventilator (MiniVent Type 845), which was set at a tidal volume of 235 µl at a rate of 105 breaths/min. A side port on the ventilator was used to supplement 100% oxygen with 2% isoflurane. The surgery was performed using a Leica surgical microscope to aid in the visualization of the mouse left anterior descending artery. The heart was accessed through a left thoracotomy and the left anterior descending artery was exposed by opening the pericardium. The suture was positioned 2 mm below the left atrial appendage to permanently ligate left anterior descending artery. The lungs were then fully inflated, and the chest wall and skin incision were sutured in layers and the thoracic cavity was closed as described^57^. Buprenorphine (sustained release (SR)-LAB, ZooPharm) was administered subcutaneously (0.5 mg/kg) at the time of surgery to provide post-operative analgesia (releases over 72 h). Mice were kept warm until recovery.

Seventy-seven mice (including C57BL6 and *Myh6*-MerCreMer::Rosa26fs-Confetti mice) were subjected to MI surgery. Of these mice 10 died between the time of surgery and 30 dpi. Fifteen post-MI mice were excluded from the study because their LVEFs, at 30 dpi, were either > 35% or < 15%.

### Echocardiography

Cardiac function was evaluated by transthoracic echocardiography performed on mice sedated with isoflurane (Piramal Critical Care) using Vevo 3100 (VisualSonics) and analyzed using the Vevo Lab software (VisualSonics). Measurements for the LVEF, end-systolic and end-diastolic volumes were calculated from the long axis as previously described^26^. Measurements for the LV PW at end-systole and end-diastole were calculated from the short axis, representing the mid-apical plane. Movies, showing LV contraction, were made using B-mode views on the parasternal long axis. Before taking images, anesthesia was adjusted to ensure heart rates of mice were between 450 and 500 beats/min.

### Quantification and statistical analysis

All data are presented as the mean ± standard error of the mean (SEM). For immunoblotting experiments 4 independent biological replicates were used in each experiment. Mice were randomized to different drug treatment groups using a preselect criteria of 15–30% LVEF. Mice with EF over or under this range were not included for drug randomization. No data points were excluded from any analysis. Most of the experiments were replicated at least once. Number of biological replicates for all experiments are mentioned in relevant figure legends. Investigators were not blinded to drug administration and outcome assessment. Statistical significance of data was determined using Grahpad Prism 9. The tests included: two-way ANOVA, followed by Sidak's multiple comparisons test; one-way ANOVA followed by Tukey's multiple comparisons test; and unpaired 2-sided Student’s t-test for comparisons involving 2 groups. F-test was used for estimation of variance when comparing two groups and Brown-Forsythe test was used for estimation of variance when multiple groups were compared using one-way ANOVA. Where described, sample size estimation was conducted using nQuery (Statsols). Differences at *P* < 0.05 were considered significant.

## Supplementary Information

Supplementary Video 1.

Supplementary Video 2.

Supplementary Video 3.

Supplementary Information 1.

## Data Availability

RNA-Seq, microarray, microRNA microarray or genome wide association studies were not performed for this manuscript. Any other data and resources generated for this manuscript are available upon reasonable request from the corresponding authors.
