# Supplementary material for: Remuscularization with triiodothyronine and β1-blocker therapy reverses post-ischemic left ventricular dysfunction and adverse remodeling
Source: Sci Rep. 2022 May 25;12:8852. doi: 10.1038/s41598-022-12723-2 (PMC9132945; doi:10.1038/s41598-022-12723-2)

Remuscularization with triiodothyronine and β_1-blocker therapy reverses post-ischemic left ventricular dysfunction and adverse remodeling

Nikolay Bogush,[1,†] Lin Tan,[1,†] Emmen Naqvi,[1] John W. Calvert,[2] Robert M. Graham,[3] W. Robert Taylor,[1,4,5] Nawazish Naqvi,[1,*] and Ahsan Husain[1,7,*]

[1]Division of Cardiology, Department of Medicine, Emory University School of Medicine, Atlanta, GA 30322, USA; [2]Department of Surgery, Carlyle Fraser Heart Center, Emory University School of Medicine, Atlanta, Georgia 30322, USA; [3]Victor Chang Cardiac Research Institute, Sydney, New South Wales 2010, Australia; [4]Atlanta Veterans Affairs Medical Center, Cardiology Division, Decatur, Georgia 30033, USA; [5]Emory University School of Medicine and Georgia Institute of Technology, Department of Biomedical Engineering, Atlanta, Georgia 30322, USA.

[†]These authors contributed equally

[*]Corresponding Authors: Ahsan Husain, PhD, Department of Medicine (Cardiology), Emory University, 3311 WMRB, 101 Woodruff Circle, Atlanta Georgia 30322. Tel: 404-727-8125; E-mail ahusai2@emory.edu or Nawazish Naqvi, PhD, Department of Medicine (Cardiology), Emory University, 323 WMRB, 101 Woodruff Circle, Atlanta Georgia 30322. E-mail nnaqvi@emory.edu

Short title: Metoprolol plus T3 dual therapy remuscularizes MI-injured hearts

Key words: Myocardial Infarction, ischemic heart failure, cardiac regeneration, thyroid hormone, β_1-adrenergic receptor blockade, metoprolol, DUSP5, cardiomyocyte proliferation.

Word Count: Abstract 148; Main Text 5,279; Methods 930

Supplementary Section includes Supplementary Methods, one Supplementary Table, six Supplementary Figures, three Supplementary videos and a separate pdf file with all the uncut immunoblotting gels.

Supplementary Methods

Antibodies and immunohistochemical studies

Immunohistochemistry was performed as detailed previously[26]. Mouse hearts were washed with PBS after harvesting, fixed in 10% formalin and then paraffin embedded and sectioned as 7 µm thick sections on Superfrost +/+ glass slides. Slides were deparaffinized by first warming in 60 ºC oven for 1 h, followed by three changes in xylene, 5 minutes each at room temperature. Sections were then rehydrated by two changes in 100% ethanol, followed by 95%, 85%, 70% ethanol and distilled water. Sodium citrate buffer was used for antigen retrieval using a protocol detailed previously[26]. Sections were blocked with 10% v/v goat serum for 30 min at room temperature before applying primary antibody. Wheat germ agglutinin ((WGA)-Alexafluor 647 conjugate (W32466, ThermoFisher) was used to identify cell borders and capillaries around the CMs. Fluorescently conjugated cardiac troponin T (cTnT) antibody (Miltenyi Biotec, 130-119-674) was used to identify CMs. Sections were washed with PBS after 1 h of incubation with primary antibodies at room temperature and mounted with glass coverslip by using GeneTex's Fluoroshield with DAPI (catalog number GTX30920). Slides were stored at 4°C until analyzed. High power (63X) images were acquired on a Leica Microsystems DM6000 microscope using the LAS X program and analyzed using Nikon's NIS Elements advanced fluorescence program. Images showing CMs in cross-sectional orientation from the mid-apical LV posterior wall (PW) were used to examine capillary-to-CM ratios by determining capillary density in a population of ~75–100 CMs. Images of CMs in cross-sectional orientation were also used to measure cross-sectional area of each CM from LV base and mid-apical region using Nikon's NIS Elements advanced fluorescence program.

Histological studies

Hearts were harvested, processed for paraffin embedding and sectioned as described above. Sections were cleared in xylene as described above before starting the trichrome staining

protocol. Sections were stained with trichrome using the trichrome stain kit (ab150686, Abcam) as previously described[26]. Trichrome stained sections were imaged using a 40x objective of Hamamatsu's NanoZoomer-SQ Digital slide scanner. Images were viewed and saved using NDP.view2 Viewing software. Viable myocardium was quantified by determining the red-stained viable cardiac muscle area in the mid-apical LV PW and dividing it by the length of the LV-mid-apical PW analyzed. Nikon's Elements 3.0 Advanced Fluorescence program was used for determining the viable myocardium and fibrosis areas. Images from all multicolor samples were acquired using a Leica SP5 confocal microscope.

CM isolation and collagenase digestion for number determination

CM number was determined after enzymatic disaggregation of the myocardium using the Langendorff heart perfusion method, as described previously[19,26]. Briefly, heparin (100–-200 µl, 1000 USP units/ml) was injected intraperitoneally eight minutes before starting the heart digestion protocol. Mice were anesthetized with 5% isoflurane and hearts harvested. After washing the heart with PBS, the aorta was cannulated for antegrade perfusion of the myocardium through the coronary circulation. Hearts were immediately perfused with cytofix (BD Biosciences, 554655) for 1 min followed by perfusion buffer (120 mM NaCl, 15 mM KCl, 0.5 mM KH_2PO_4, 5 mM $NaHCO_3$, 10 mM HEPES, and 5 mM glucose, at pH 7.0) for 2 minutes at 37 °C using the Langendorff perfusion system. After washing for 2 min, the hearts were perfused with perfusion buffer containing collagenase type 2 (Worthington, LS004176) for 10–15 min at 37 °C. Collagenase concentration was 2 mg/ml. After ~12 min of digestion, the cardiac ventricles placed in a dish containing STOP buffer (perfusion buffer plus 10% bovine calf serum). The ventricles were teased apart into small pieces followed by trituration through pipettes of progressively smaller diameters. The digested CMs from each heart were collected in a 15 ml falcon tube and more STOP buffer was added to a volume of 10 ml. The final cell suspension was used to count CMs using a hemocytometer. CMs were not further purified to prevent losses. Further purification is not

required because CMs are clearly identifiable (by phase contrast microscope) because of their cytoplasmic size and rod shape[18]. Four aliquots from each digested heart were counted by an experienced investigator and the mean value was used to establish the total number of ventricular CMs in that heart. The same investigator measured CM number for all studies presented to minimize variability. Optimization of this protocol to achieve maximal digestion efficiencies (between 97% and 99%) is detailed elsewhere[18].

Immunoblotting

For immunoblotting, hearts were enzymatically digested and CMs purified (>95% purity) as described previously[18]. Aliquots of CMs were snap frozen in liquid nitrogen and stored at –80 °C until used for immunoblotting. For immunoblotting, whole cell CM lysates were generated by re-suspending CMs in 250 μl of RIPA buffer (Cell Signaling, 9806S) supplemented with phosphatase inhibitor cocktail 2 and 3 (Sigma-Aldrich, P5726-1ML and P0044-1ML), 0.1 mM phenylmethylsulfonyl fluoride (PMSF, Sigma-Aldrich, 93482-50ML-F) and protease inhibitor cocktail (Roche, 11697498001). The lysates were centrifuged to remove particulates and the supernatants were snap-frozen in liquid nitrogen. The samples were analyzed using SDS–polyacrylamide gel (12–18%) electrophoresis as previously described[19]. Proteins thus resolved were transferred to a PVDF membrane and probed with GAPDH antibody. Based on GAPDH, loading of each sample was adjusted so that all samples contained an equal amount of GAPDH. Membranes were probed with DUSP5 antibody (ab200708, Abcam). This DUSP5 antibody was previously shown to specifically detect DUSP5[19]. For quantitative analysis, the membrane was then stripped and re-probed with GAPDH to ensure that loading was normalized for each sample. Other antibodies used in this study are detailed in Supplementary Table S1; they were target validated either by Cell Signaling or Abcam or by us using siRNA knockdown studies. ImageLab program (Bio-Rad) was used for quantification.

Supplementary Table S1. Information about the antibodies used for immunoblotting.

**Antibody**	**Vendor**	**Catalog number**
β_1-adrenergic receptor	Cell Signaling	12271
Cyclin A2	Abcam	ab181591
Cyclin B1	Abcam	ab32053
Cyclin D1	Abcam	ab134175
DUSP5	Abcam	ab200708
ERK1/2	Cell Signaling	4695
GAPDH	Cell Signaling	2118
MEK1/2	Cell Signaling	9126
Phospho-ERK1/2 (T202/Y204)	Cell Signaling	4370
Phospho-MEK (S217/221)	Cell Signaling	9121
ECT2	Cell Signaling	52021

These antibodies are profiled in 1DegreeBio and many were validated by us using genetic knockout models or siRNA.

Supplementary Figures with Figure Legends

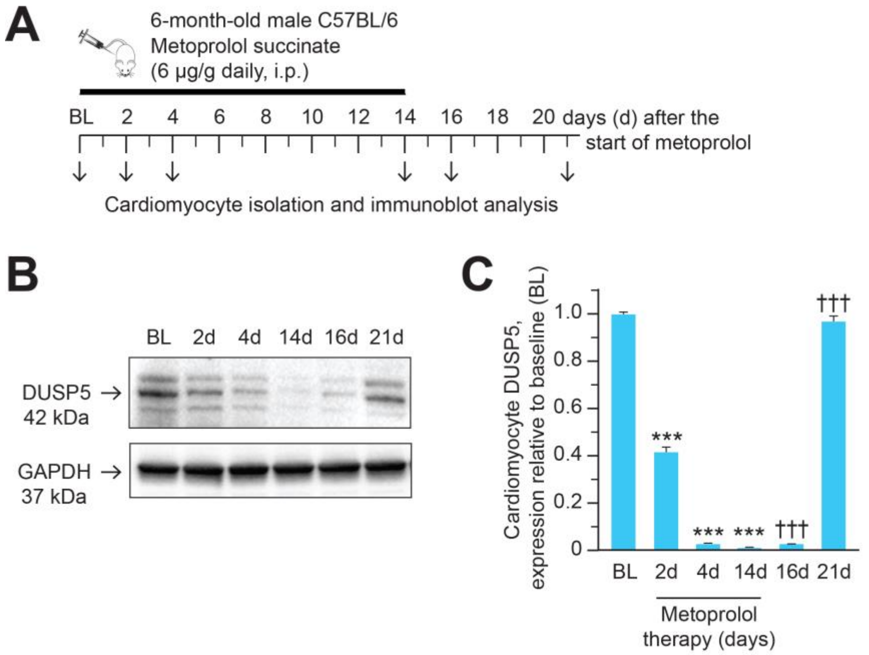

Supplementary Figure S1. Metoprolol reversibly inhibits CM DUSP5 expression. (**A**) Schematic of the metoprolol dosing schedule and the times at which hearts were collected to determine LV CM DUSP5 levels (arrows). The period over which metoprolol therapy was given is indicated by the bar. BL, baseline. (**B**) Immunoblots (representative of 4 biological replicates) showing DUSP5 and GAPDH expression in CMs. Usually a single band at 42 kDa is prominently observed when DUSP5 expression is analyzed in snap-frozen mouse heart tissue; this band is absent in heart tissue obtained from DUSP5-specific siRNA treated mice[19] indicating antibody specificity. The process of enzymatic heart disaggregation and CM purification, which takes several minutes, leads to the generation of DUSP5 degradation- and post-translational modification-products, which can be seen above and below the unmodified 42 kDa product. (**C**) Bar graph showing CM DUSP5 expression (only the native 42 kDa band shown in B, was evaluated) before metoprolol therapy (BL), during metoprolol therapy (2d–14d) and after withdrawal of therapy at the end of day-14. The data are shown as the mean ± SEM; $n = 4$ biological replicates. Comparisons were made by 1-way ANOVA followed by Tukey's multiple comparisons test. ***$p < 0.001$ compared with BL values and $^{†††}p < 0.001$ compared with values on day 14.

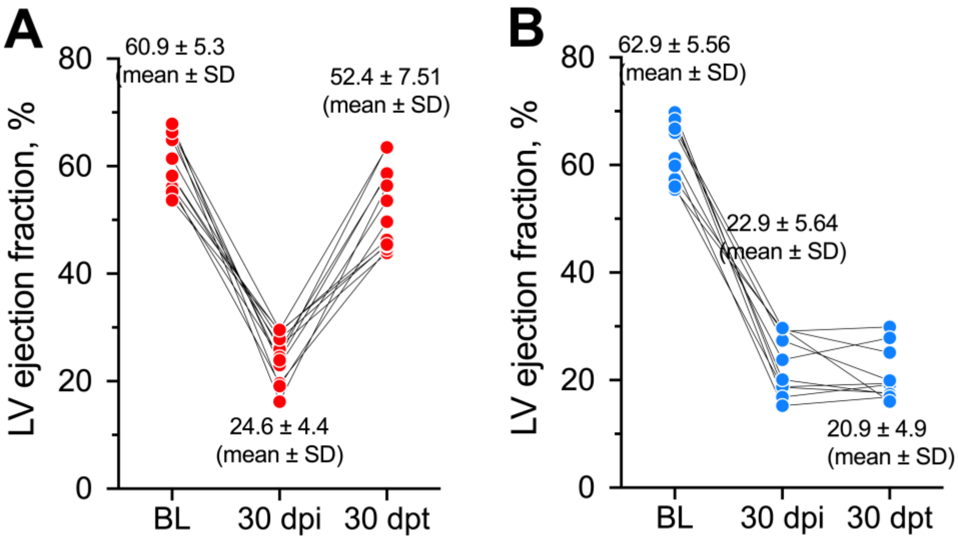

Supplementary Figure S2. Pilot study for estimating the degree of effect of M+T3 therapy on LVEF of 30 post-MI hearts. (**A**) Effect of M+T3 therapy on LVEF at 30 dpt on post-MI hearts. (**B**) Effect of metoprolol monotherapy on LVEF at 30 dpt on post-MI hearts. Results are shown only for mice in which MI injury resulted in LVEFs between 15% and 30%. In (A, B) results are shown only for mice in which MI injury at 30 dpi resulted in LVEFs between 15% and 30%. Mean ± SD are also indicated. BL, baseline. The experimental protocol is shown in Fig. 3A.

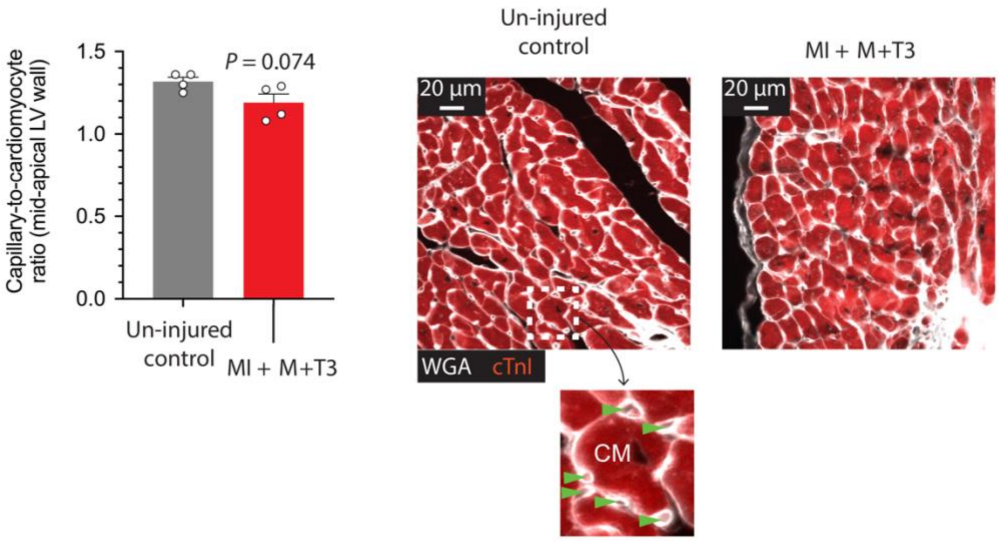

Supplementary Figure S3. Capillary-to-CM ratio in the LV mid-apical myocardium of chronic post-MI hearts after metoprolol-and-T3 therapy. Comparisons of post-MI hearts 150 days after therapy and uninjured age-matched control hearts were made by a 2-tailed t-test. Representative images of immunohistochemically stained heart sections where CMs were identified using cardiac troponin T (cTnT, red) and cell boundaries were labeled with wheat germ agglutinin (WGA, white), which was also used to identify capillaries (green arrowheads, inset) that surround CMs (inset). (*n* = 4 mice per group). Data are shown as individual values and the mean ± SEM.

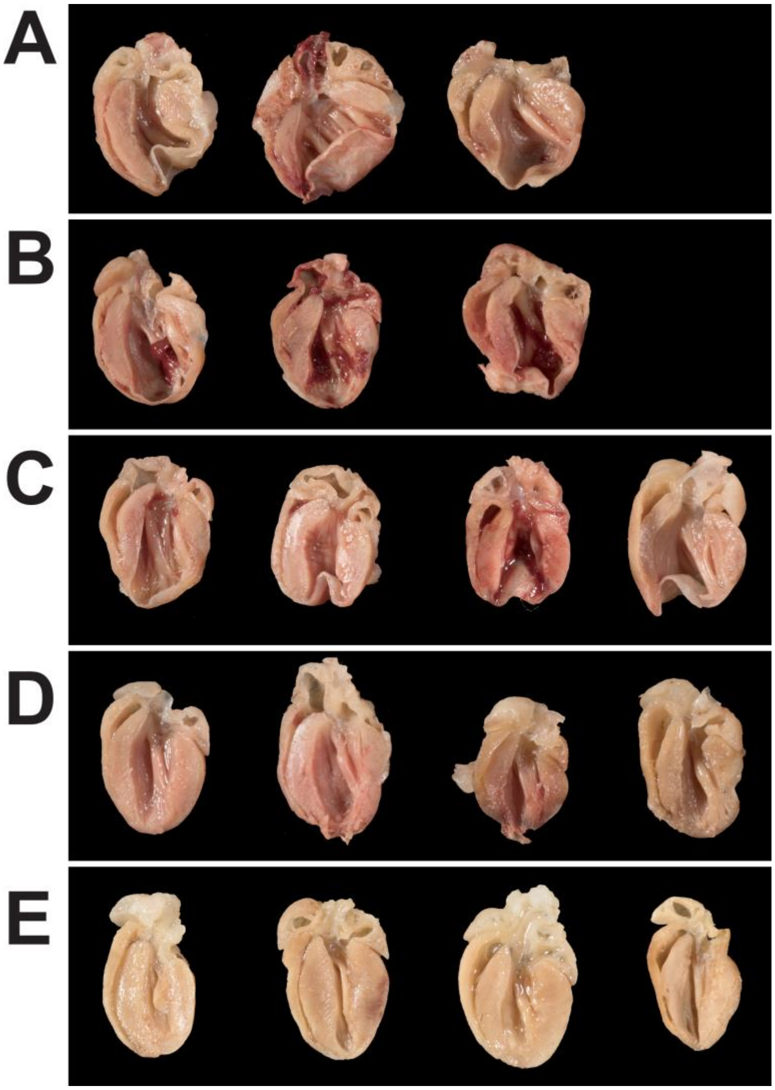

Supplementary Figure S4. Gross morphology of post-MI hearts after M+T3 therapy or monotherapy with metoprolol or T3 and uninjured control hearts. (**A–D**) Examples of chronic post-MI hearts at 150 dpt with no therapy (A), T3 monotherapy (B), metoprolol monotherapy (C) or M+T3 therapy (D). (**E**) The hearts of age-matched uninjured controls. The hearts second from the left in panels a, d and e are also shown in Fig. 6A. In these images, the hearts are longitudinally cut, and the halves depicted are those with the LV positioned on the right.

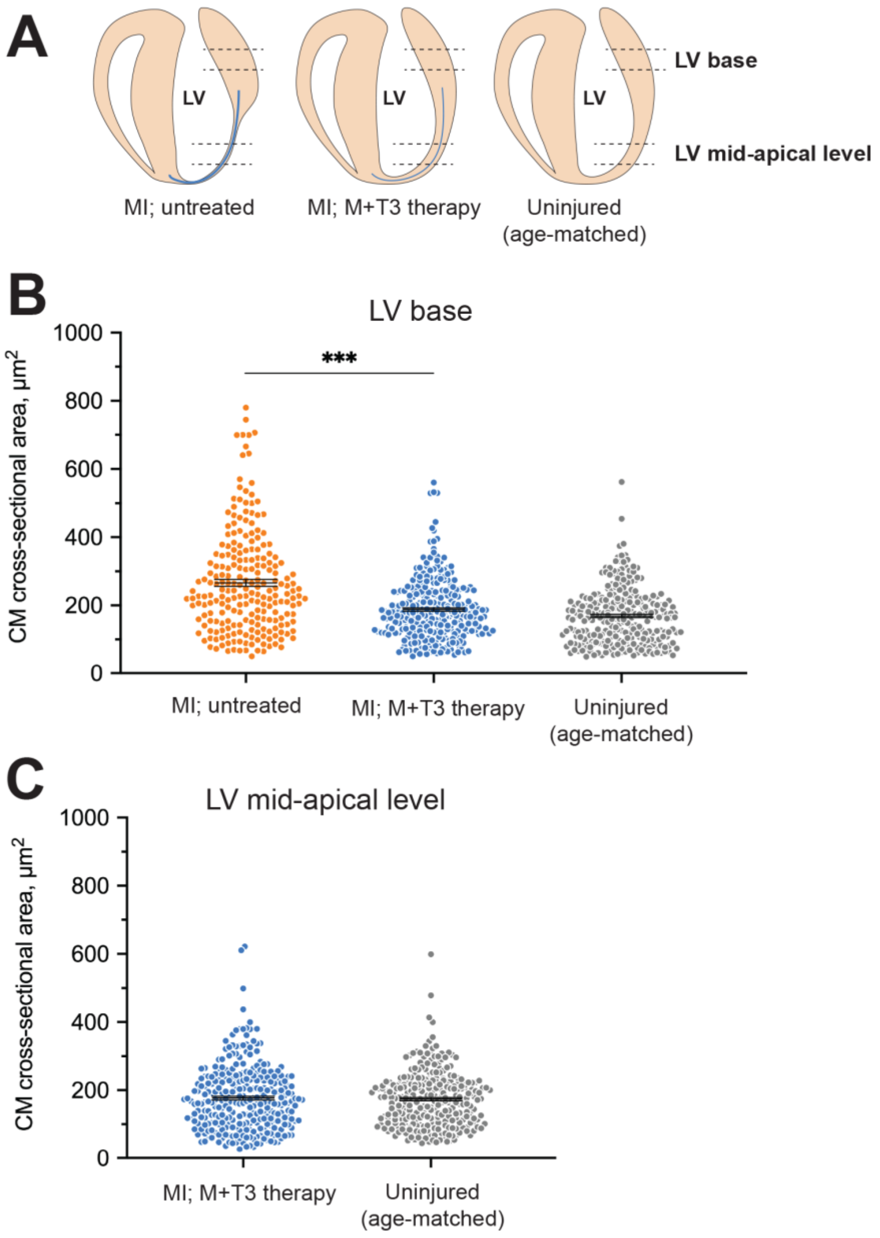

Supplementary Figure S5. MI injury-induced cardiac hypertrophy is blocked by M+T3 therapy. (**A**) Illustrations depicting the gross structure of a post-MI heart (left), a post-MI heart after M+T3 therapy (middle) and an uninjured age-matched control heart (right). Approximate locations of the remote zone (LV base) and the LV mid-apical PW, which were sampled in histological sections for the estimation of CM cross-sectional areas. (**B, C**) Size of CMs in the LV base (B) and LV mid-apical PW (C). *n* = 3–4 mice per group. Data are shown as individual values and the mean ± SEM. ***p < 0.001.

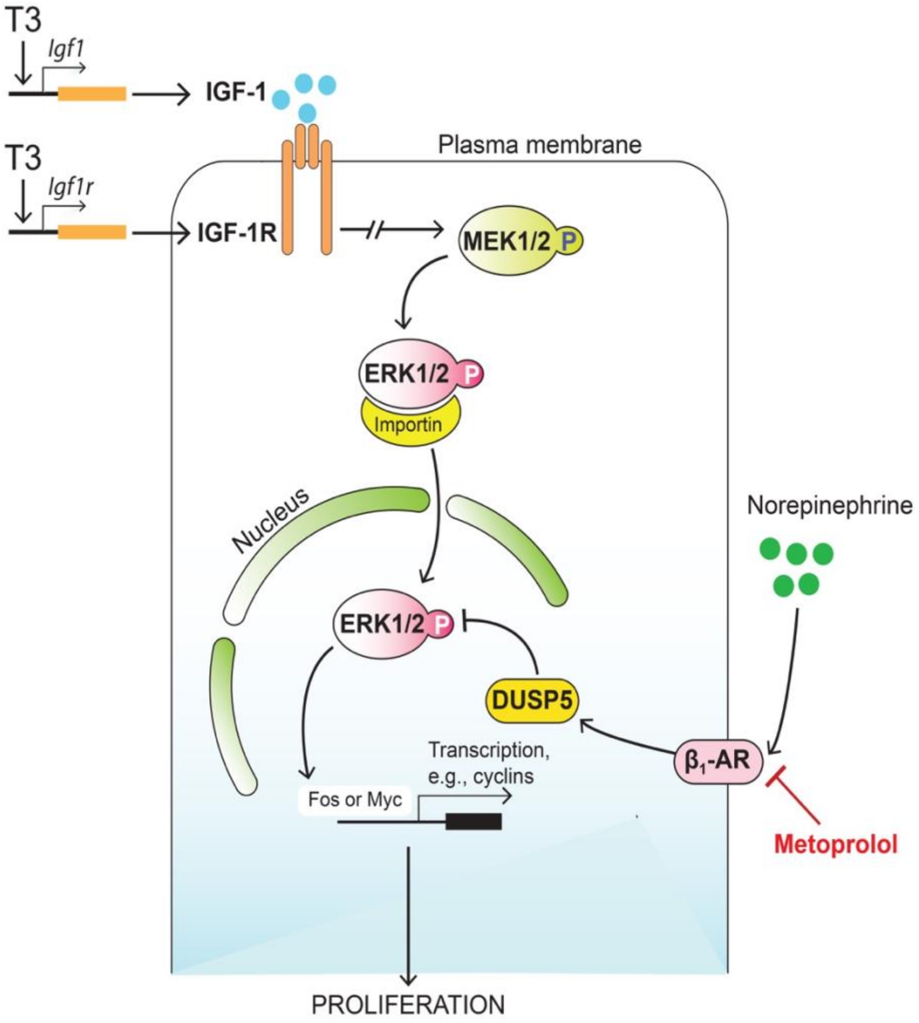

Supplementary Figure S6. A working model showing how metoprolol (M) + T3 therapy stimulates proliferation in deeply quiescent adult cardiomyocytes. IGF-1 and IGF-1 receptor (IGF-1R) expression by T3 and subsequent activation of IGF-1R/ ERK1/2 signaling results in CM proliferation in neonatal mice. In adult mice, however, exogenous T3 does not stimulate CM proliferation despite robustly increasing IGF-1/IGF-1R signaling. This failure results from a developmental increase in a nuclear p-ERK1/2-specific dual specificity phosphatase-5 (DUSP5) in CMs. This developmental increase in CM DUSP5 expression is controlled by β1-AR signaling. Acute blockade of β1-AR signaling using cardioselective metoprolol depletes DUSP5 and induces CM proliferation when combined with T3 muscularizing adult heart.

Supplementary Videos

Supplementary Video S1. Mouse transthoracic echocardiogram showing parasternal long axis view of the heart of a representative mouse at baseline. This video is No. 1 of 3 videos obtained from mouse I.D. No.: 476. This movie is representative of those from 3 other mice.

Supplementary Video S2. Mouse transthoracic echocardiogram showing parasternal long axis view of the heart of a representative mouse at 30 days post-MI injury. This video is No. 2 of 3 videos obtained from mouse I.D. No.: 476. This video is representative of those from 3 other mice.

Supplementary Video S3. Mouse transthoracic echocardiogram showing parasternal long axis view of the heart of a representative mouse at 150 days after M+T3 therapy. This video is No. 3 of 3 videos obtained from mouse I.D. No.: 476. This video is representative of those from 3 other mice.

Bogush et al., Figure 1B

β1 Adrenergic Receptor 50kDa

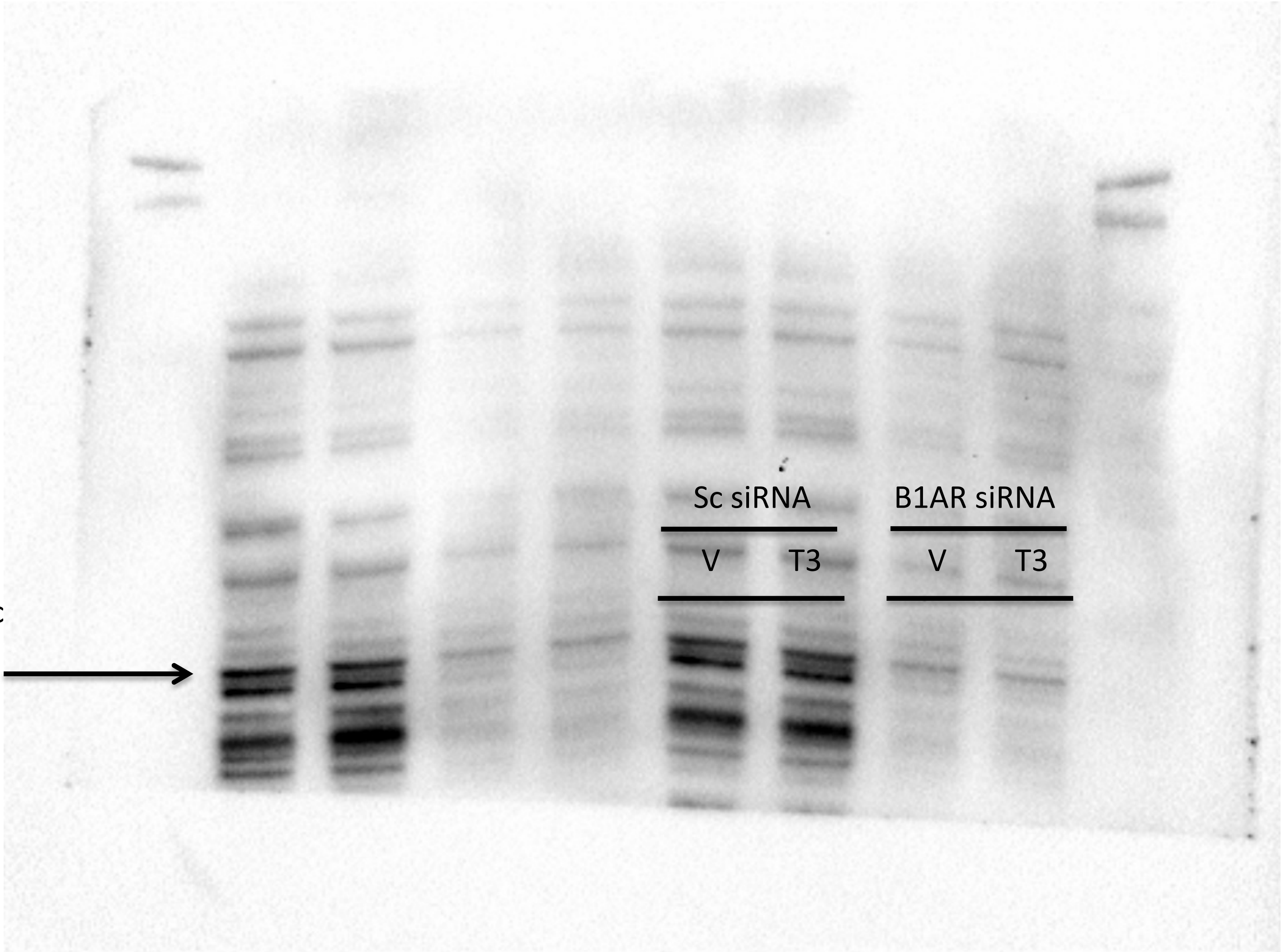

Bogush et al., Figure 1B

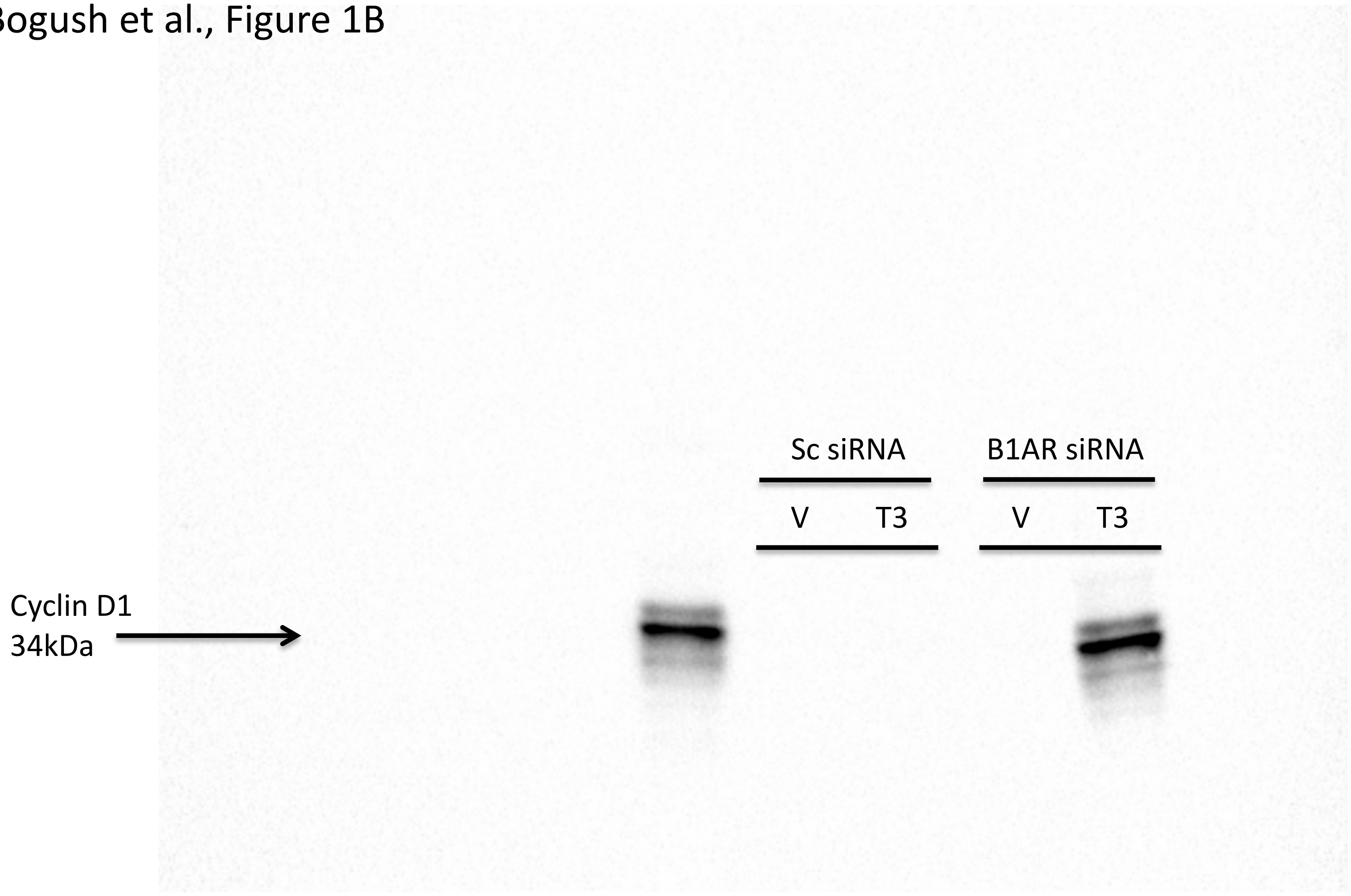

Bogush et al., Figure 1B

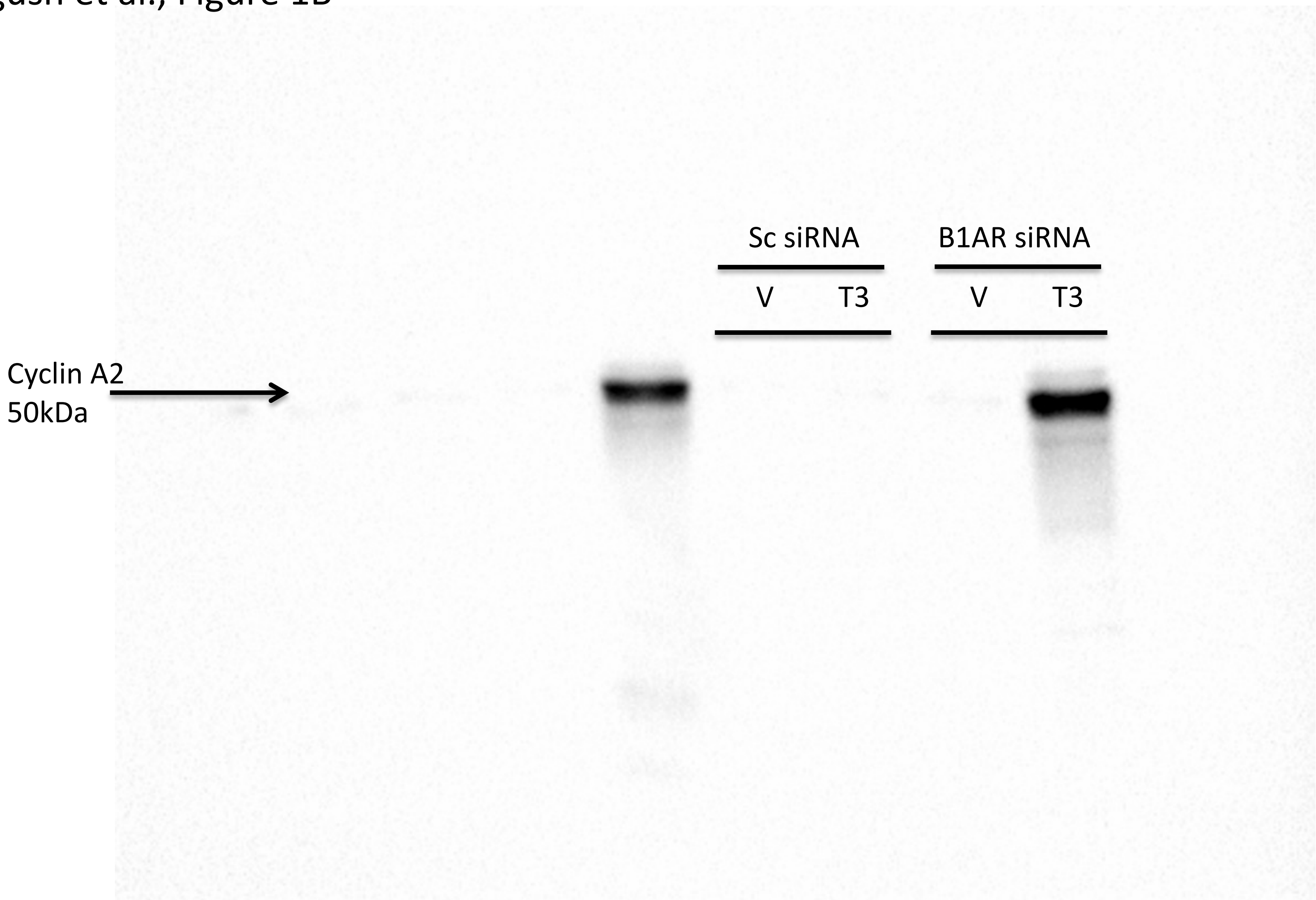

Bogush et al., Figure 1B

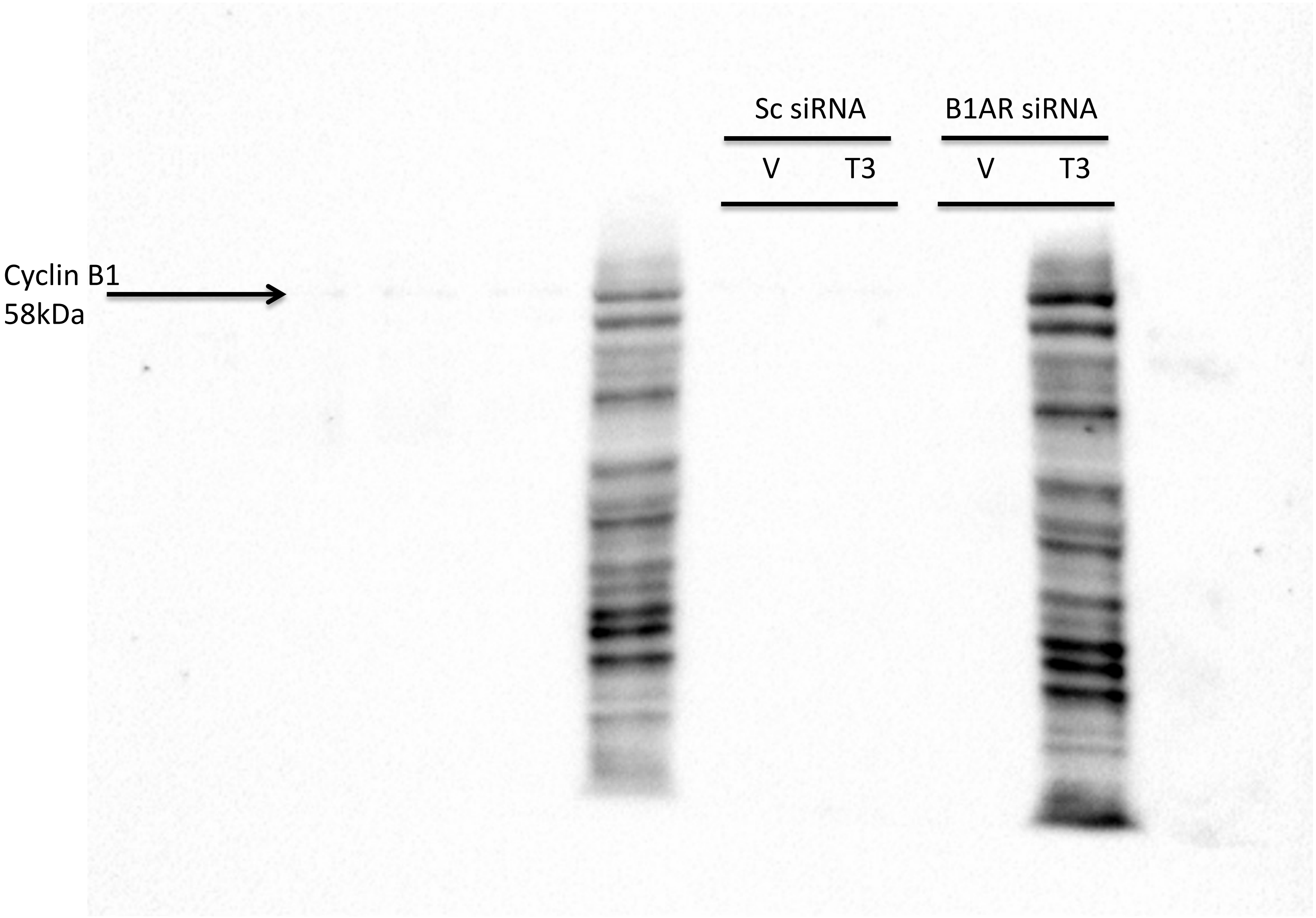

V T3 V T3

Bogush et al., Figure 1B

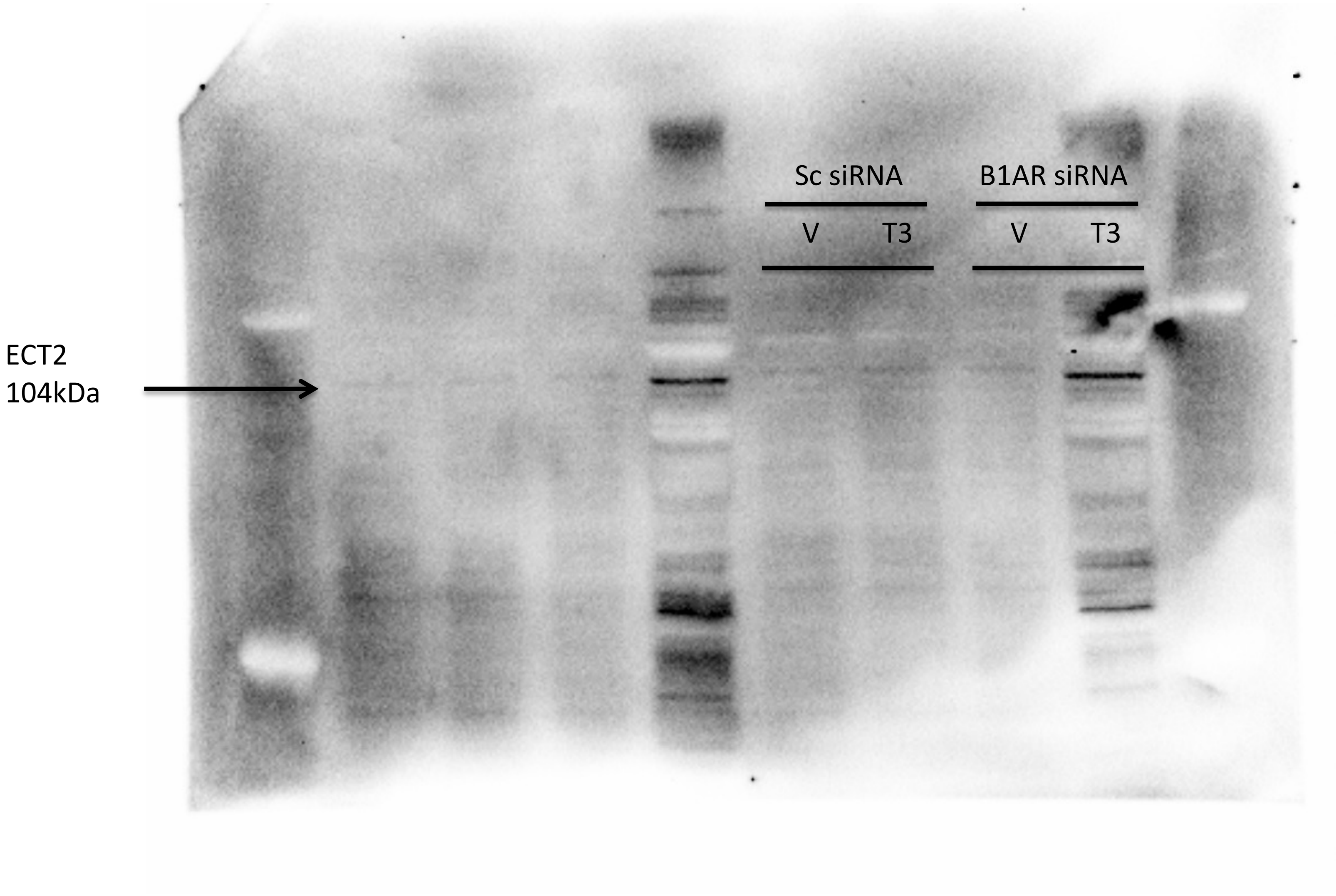

Bogush et al., Figure 1B

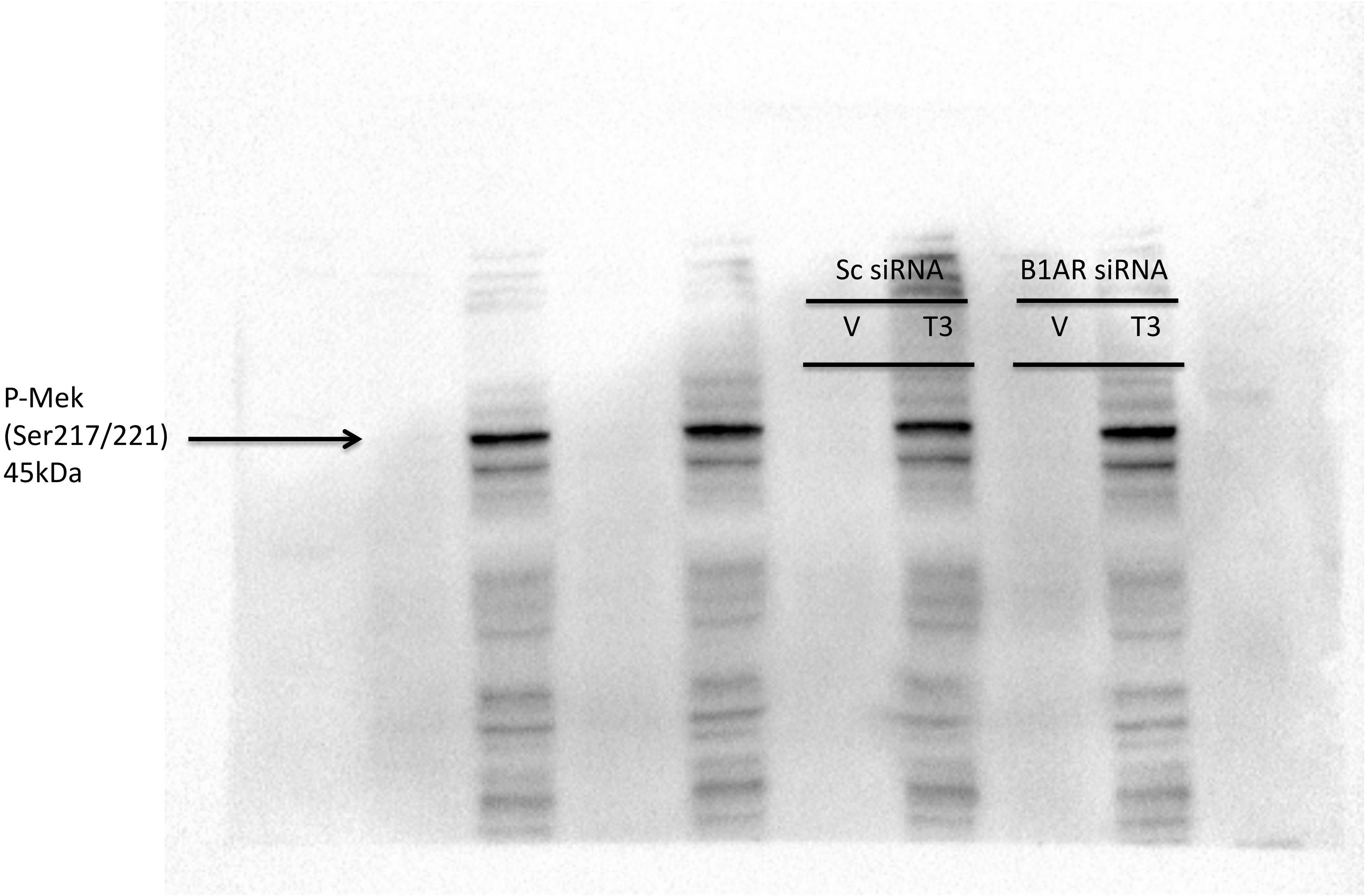

Bogush et al., Figure 1B

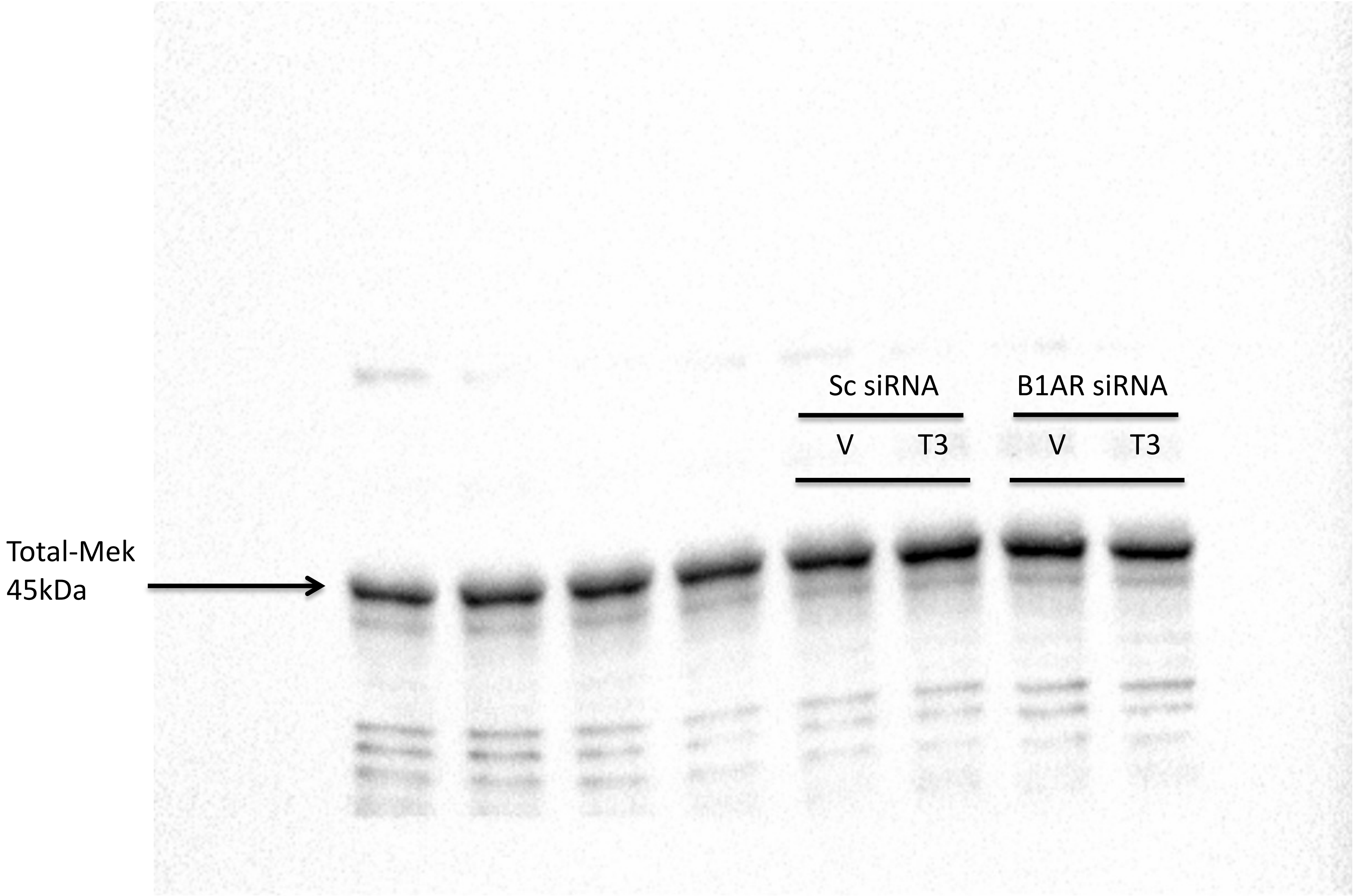

Bogush et al., Figure 1B

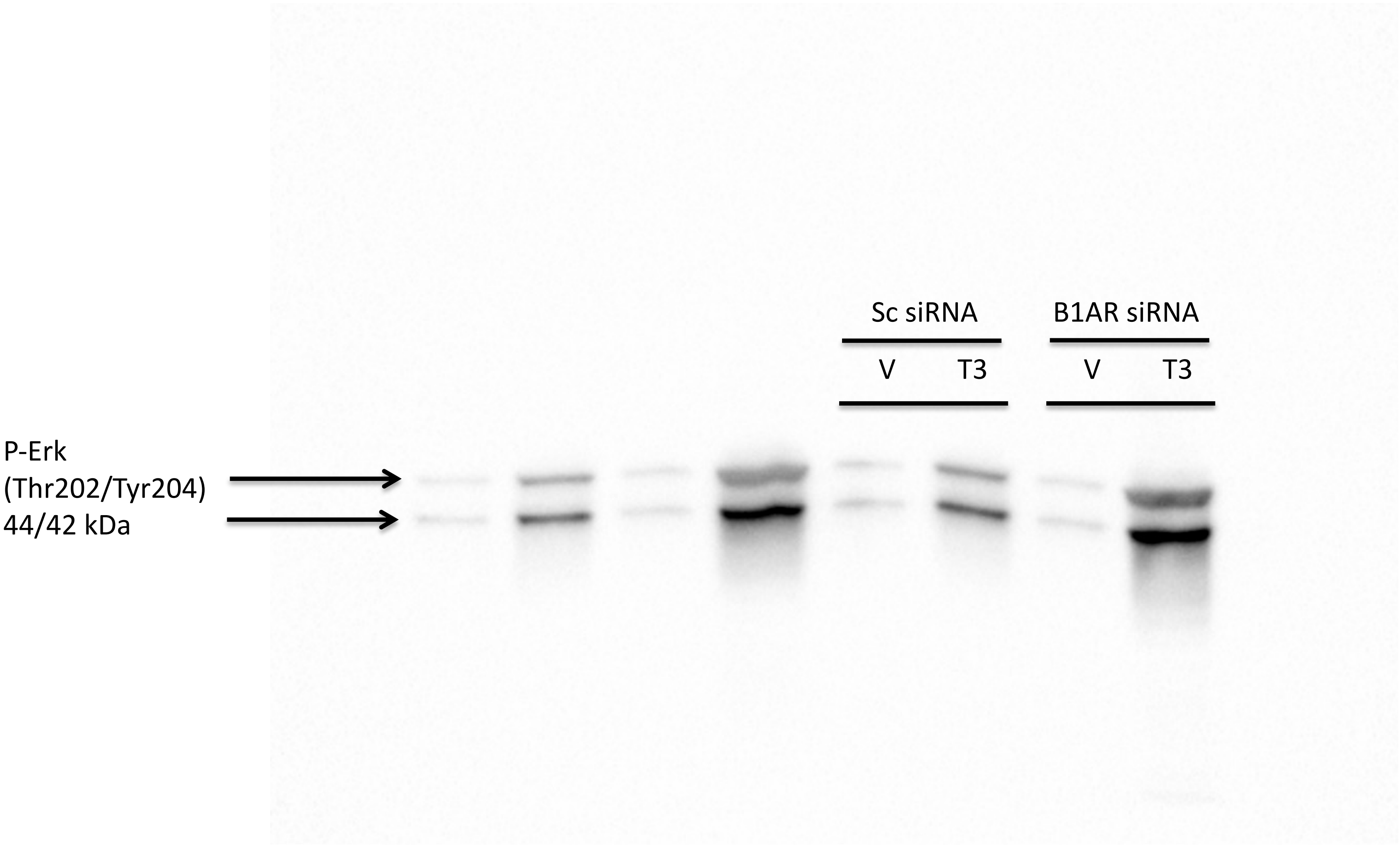

Bogush et al., Figure 1B

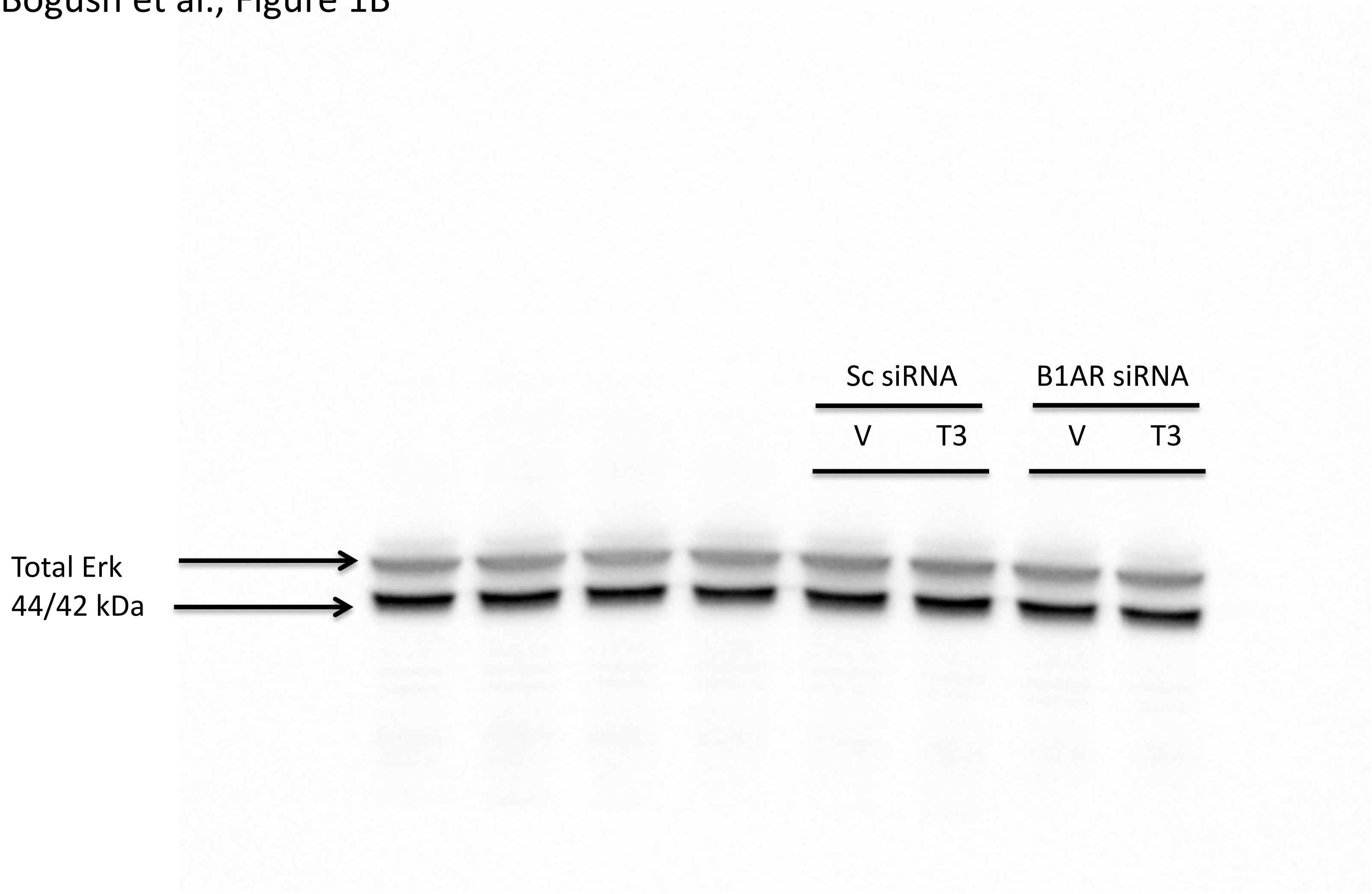

Bogush et al., Figure 1B

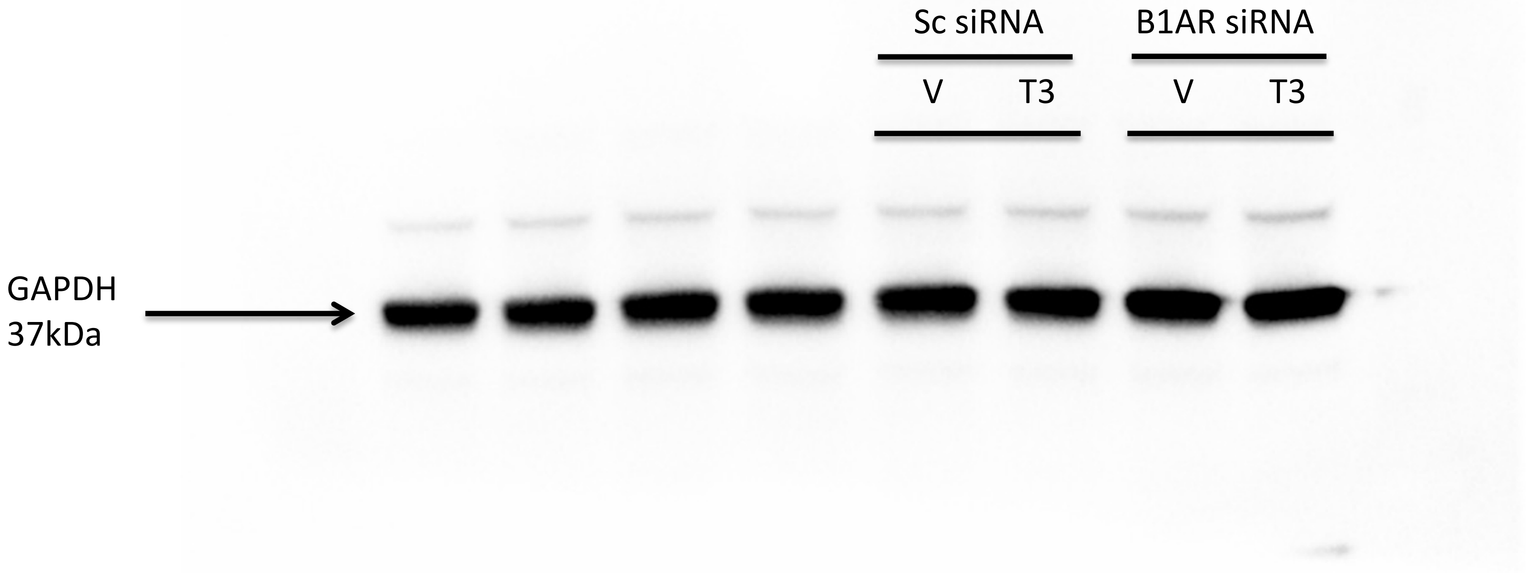

Bogush et al., Figure S1B

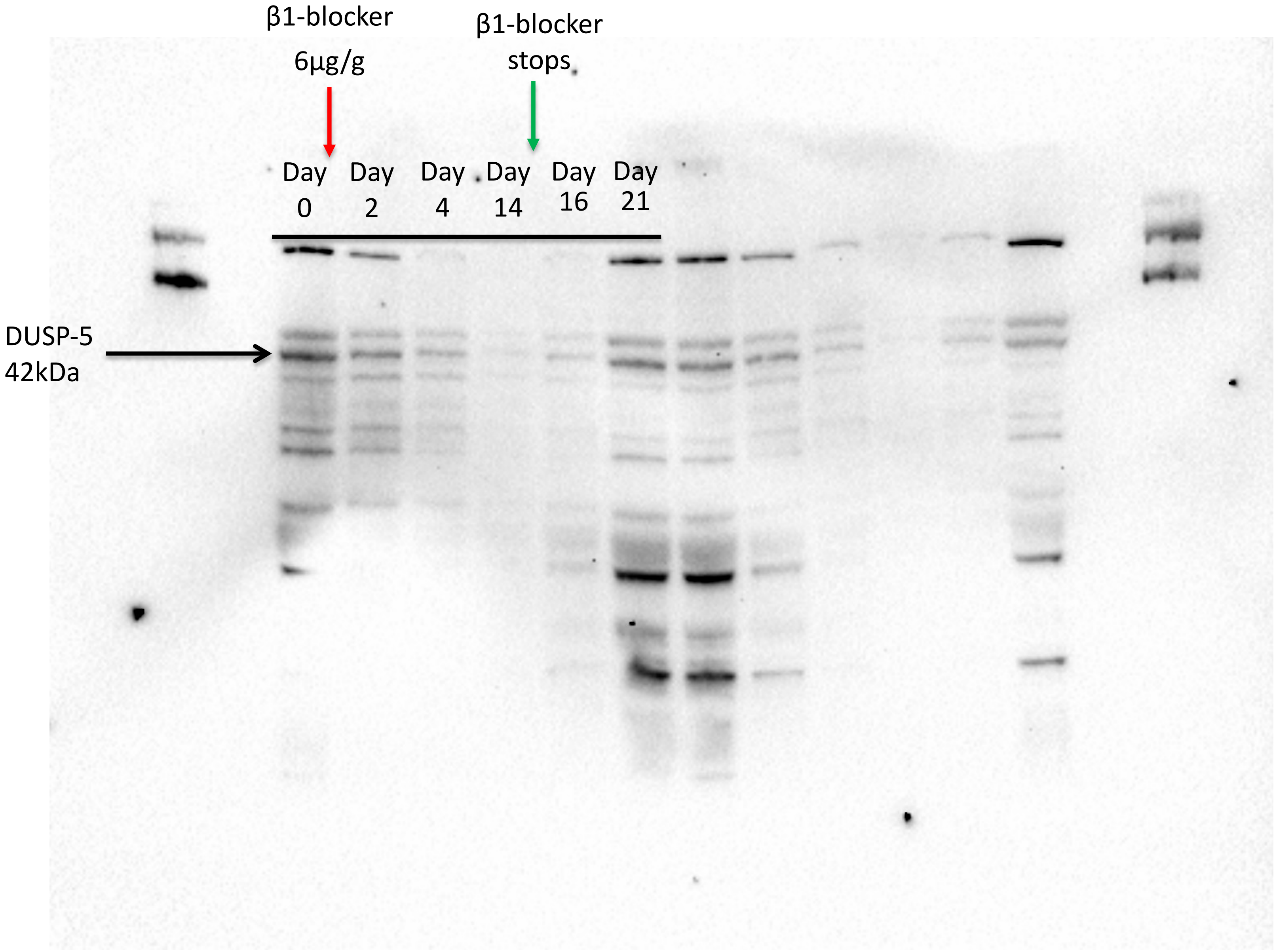

Bogush et al., Figure S1B

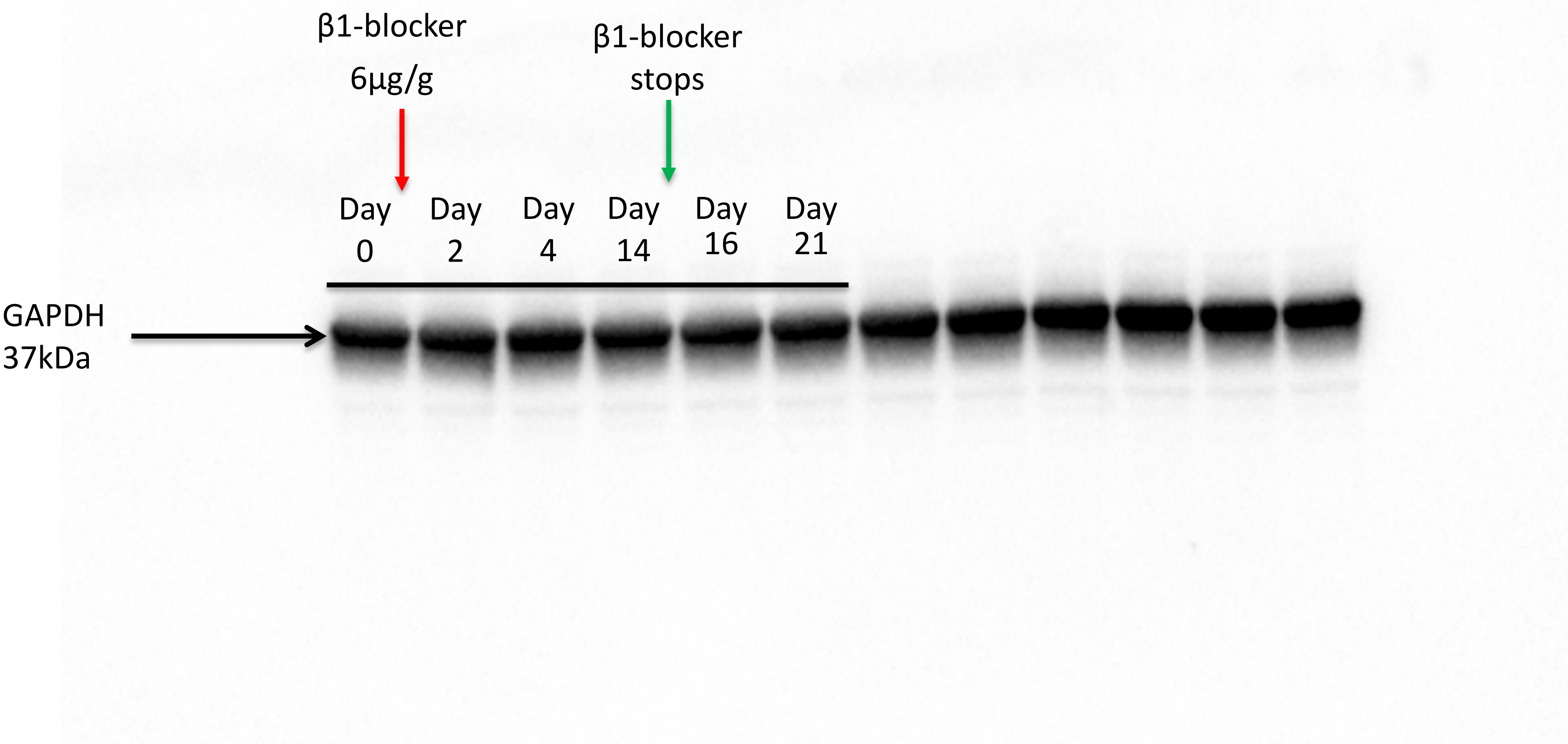

Supplement: Supplementary file 4 — Supplementary Information 1. [file 41598_2022_12723_MOESM4_ESM.pdf]
